# Targeting Oncogenic Src Homology 2 Domain-Containing Phosphatase 2 (SHP2) by Inhibiting Its Protein–Protein Interactions

**DOI:** 10.1021/acs.jmedchem.1c01371

**Published:** 2021-10-29

**Authors:** Sara Bobone, Luca Pannone, Barbara Biondi, Maja Solman, Elisabetta Flex, Viviana Claudia Canale, Paolo Calligari, Chiara De Faveri, Tommaso Gandini, Andrea Quercioli, Giuseppe Torini, Martina Venditti, Antonella Lauri, Giulia Fasano, Jelmer Hoeksma, Valerio Santucci, Giada Cattani, Alessio Bocedi, Giovanna Carpentieri, Valentina Tirelli, Massimo Sanchez, Cristina Peggion, Fernando Formaggio, Jeroen den Hertog, Simone Martinelli, Gianfranco Bocchinfuso, Marco Tartaglia, Lorenzo Stella

**Affiliations:** † Department of Chemical Science and Technologies, University of Rome Tor Vergata, Rome 00133, Italy; ‡ Genetics and Rare Diseases Research Division, Ospedale Pediatrico Bambino Gesù, IRCCS, Rome 00146, Italy; § Dipartimento di Oncologia e Medicina Molecolare, Istituto Superiore di Sanità, Rome 00161, Italy; ∥ Institute of Biomolecular Chemistry, Padova Unit, CNR, Padova 35131, Italy; ⊥ Hubrecht institute-KNAW and University Medical Center Utrecht, Utrecht 3584 CT, The Netherlands; # Department of Chemical Sciences, University of Padova, Padova 35131, Italy; @ Centre of Core Facilities, Istituto Superiore di Sanità, Rome 00161, Italy; ∇ Institute of Biology Leiden, Leiden University, Leiden 2333 BE, The Netherlands

## Abstract

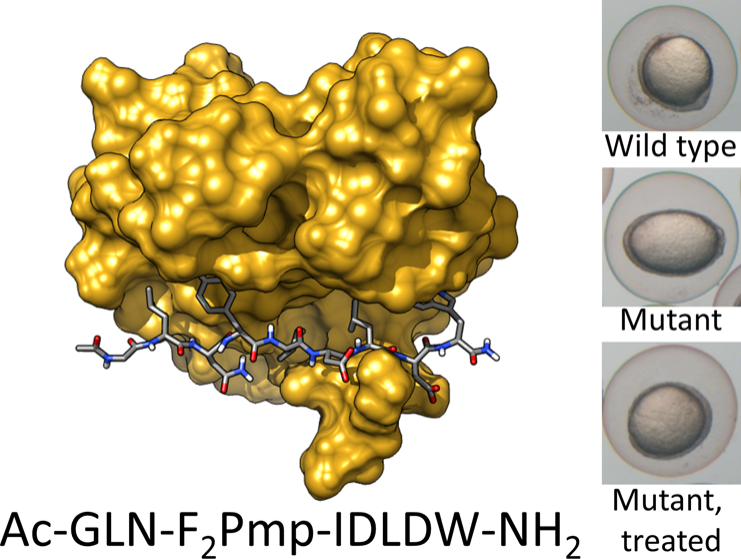

We developed a new class of inhibitors of protein–protein interactions of the SHP2 phosphatase, which is pivotal in cell signaling and represents a central target in the therapy of cancer and rare diseases. Currently available SHP2 inhibitors target the catalytic site or an allosteric pocket but lack specificity or are ineffective for disease-associated SHP2 mutants. Considering that pathogenic lesions cause signaling hyperactivation due to increased levels of SHP2 association with cognate proteins, we developed peptide-based molecules with nanomolar affinity for the N-terminal Src homology domain of SHP2, good selectivity, stability to degradation, and an affinity for pathogenic variants of SHP2 that is 2–20 times higher than for the wild-type protein. The best peptide reverted the effects of a pathogenic variant (D61G) in zebrafish embryos. Our results provide a novel route for SHP2-targeted therapies and a tool for investigating the role of protein–protein interactions in the function of SHP2.

## Introduction

The Src homology 2 (SH2) domain-containing phosphatase 2 (SHP2), encoded by the *PTPN11* gene,^1^ is ubiquitously expressed and mediates signal transduction downstream of various receptor tyrosine kinases (RTKs). This phosphatase is required for full and sustained activation of the RAS/MAP kinase pathway^2^ and modulates signaling also through the PI3K-AKT and JAK-STAT pathways, among others. SHP2 is involved in the regulation of multiple cell processes, including proliferation, survival, differentiation, and migration, and its functional upregulation contributes to oncogenesis and underlies developmental disorders.^1^ Somatically acquired, gain of function mutations in *PTPN11* are the major cause of juvenile myelomonocytic leukemia (JMML),^3^ a rare and aggressive myelodysplastic/myeloproliferative disorder of early childhood with a very poor prognosis, for which no drugs are presently available. Somatic *PTPN11* mutations also occur in childhood myelodysplastic syndromes, acute monocytic leukemia (AMoL, FAB M5), and acute lymphoblastic leukemia (ALL, “common” subtype).^3,4^ More rarely, activating mutations in this gene are found in adult myelodysplastic syndromes, chronic myelomonocytic leukemia, and solid tumors, including neuroblastoma, glioma, embryonal rhabdomyosarcoma, lung cancer, colon cancer, and melanoma. In addition to malignancies driven by *PTPN11* mutations, several forms of cancer are linked to the activity of wild-type SHP2. SHP2 is required for survival of RTK-driven cancer cells,^5^ is a central node in intrinsic and acquired resistance to targeted cancer drugs,^6^ and plays a role as a mediator of immune checkpoint pathways^7,8^ and of induction of gastric carcinoma by *Helicobacter pylori*.^9,10^

In addition to its role in cancer, SHP2 is involved in two disorders that belong to a family of rare diseases collectively known as RASopathies. Germline missense mutations in *PTPN11* occur in ∼50% of individuals affected by Noonan syndrome (NS),^11^ one of the most common nonchromosomal disorders affecting development and growth,^12^ and in ∼90% of patients affected by the clinically related Noonan syndrome with multiple lentigines (NSML, formerly known as LEOPARD syndrome).^13,14^ RASopathies are characterized by congenital cardiac anomalies, hypertrophic cardiomyopathy, short stature, musculoskeletal anomalies, facial dysmorphisms, variable intellectual disability, and susceptibility to certain malignancies.^15^ To date, the only treatment in use for NS and related disorders is growth hormone therapy to improve linear growth.^12^

**Figure 1:**
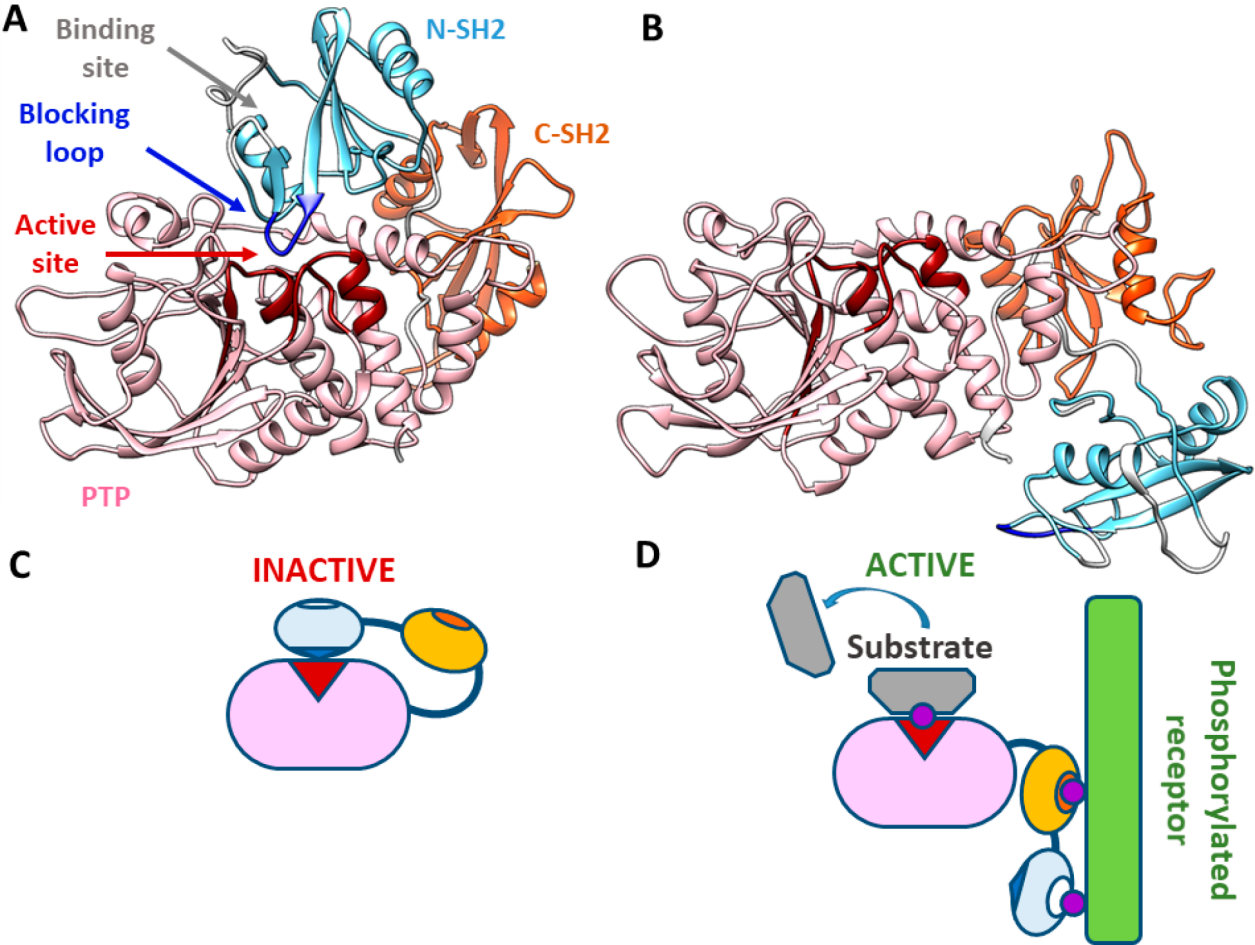
SHP2 structure and scheme of the activation process. (A) Crystallographic structure of the closed, autoinhibited state of SHP2 (PDB entry 2SHP). The active site (red) of the PTP domain (pink) is blocked by the N-SH2 domain (light blue) and particularly by its blocking loop (DE loop, blue). Access to the binding site of the N-SH2 domain is blocked by two loops (EF and BG, white). The C-SH2 domain is colored orange. (B) Crystallographic structure of the open, active state of SHP2 (PDB entry 6CRF). With respect to the autoinhibited state, the N-SH2 domain moves to the other side of the PTP domain, freeing the active site. At the same time, the EF and BG loops open and the N-SH2 binding site is accessible. Segments missing in the experimental structures were modeled as previously described.^21^ (C) Schematic model of the autoinhibited state. Under basal conditions, the protein is autoinhibited by the N-SH2 domain, blocking the active site. (D) Schematic model of the active state. Upon interaction with binding partners, an open, active conformation is stabilized.

The structure of SHP2 includes two Src homology 2 (SH2) domains, called N-SH2 and C-SH2, followed by the catalytic PTP domain, and an unstructured C-terminal tail (Figure 1).^1,16^ SH2 domains are recognition elements that bind protein sequences containing a phosphorylated tyrosine (pY).^17,18^ In SHP2, they mediate association with RTKs, cytokine receptors, cell adhesion molecules, and scaffolding adaptors. Therefore, SHP2 (together with the closely related SHP1) is recruited (through its SH2 domains) by motifs containing two pYs and dephosphorylates other (or even the same) pYs through its PTP domain.

Under basal conditions, the N-SH2 domain blocks the active site of the PTP domain, inserting a loop (DE or “blocking” loop) into the catalytic pocket;^16^ consistently, the basal activity of SHP2 is very low. Association of SHP2 with its binding partners through the SH2 domains favors the release of this autoinhibitory interaction, making the catalytic site available to substrates and causing activation (Figure 1).^19−23^ Specifically, structures of the N-SH2 domain associated with phosphopeptide sequences show that association with binding partners induces a conformational change in the blocking loop, which loses complementarity to the active site.^24^ At the same time, the N-SH2/PTP interaction allosterically controls the conformation of the N-SH2 domain binding site. Structures of the autoinhibited protein show that the binding site of the N-SH2 domain is closed by two loops (EF and BG). By contrast, in structures of the isolated N-SH2 domain,^24^ or the recently reported structure of the active state of SHP2,^23^ the binding site is open (Figure 1). Consequently, we and others have hypothesized that the transition between the closed, autoinhibited state and the open, active conformation is coupled to an increased affinity for binding partners.^19,21−23^

The spectrum of pathogenic *PTPN11* mutations is generally consistent with this picture of SHP2 regulation. Most mutations cluster at the N-SH2/PTP interface, destabilizing the interaction between these two domains and causing constitutive activation of the phosphatase.^19−21^ These mutations concomitantly induce an increased responsiveness to activation by association of bisphosphorylated sequences with the SH2 domains.^19,21−23^ Other mutations localize in the binding site of the SH2 domains and simply cause an increased affinity for phosphorylated binding partners.^20^ In all cases, the final effect is an upregulation of the RAS/MAPK signal transduction pathway.

All of the findings reported above clearly indicate SHP2 as an important molecular target for cancer and RASopathies.^25,26^ Research efforts in SHP2-targeted drug discovery have long been focused mainly on active-site inhibitors,^27,28^ which however are affected by a lack of target specificity.^26,27^^29^

Allosteric inhibitors stabilizing the autoinhibited structure of SHP2 by binding to a pocket located at the interdomain interface in the closed conformation of the phosphatase represent a recent, alternative pharmacological strategy.^5,25,26,30−35^ SHP099, an inhibitor developed by Novartis, is finding promising applications in the treatment of RTK-driven cancers^33^ and in combined therapy against drug resistant cells.^6^ However, allosteric inhibitors are generally poorly effective in the case of activating *PTPN11* mutants, because their binding site is lost in the open conformation of the enzyme.^23,25^

Due to the allosteric mechanism described above, SHP2 activation and its association with binding partners are coupled events. Therefore, the effect of NS- and leukemia-causing mutations destabilizing the autoinhibited conformation is twofold: they cause an increase in the phosphatase activity of the protein but at the same time favor the N-SH2 conformation suitable for binding phosphorylated proteins, thus increasing the overall responsiveness of SHP2 to its interaction partners. Several lines of evidence indicate that the second event, rather than the enhanced basal activity, is essential for the abnormal activation of the RAS/MAPK pathway. Some pathogenic variants, such as the NS-associated p.T42A, simply increase the binding affinity of the N-SH2 domain, without causing basal activation;^19,22^ in addition, the ability of SHP2 to associate with binding partners is preserved in all of the disease-associated *PTPN11* mutations.^20,36,37^ Truncated constructs with deletion or partial deletion of the N-SH2 domain cause a dramatic increase in the enzymatic activity of SHP2 and, at the same time, a complete loss of its ability to bind signaling partners. These constructs do not affect development in heterozygous mice^2^ and do not cause any aberrant phenotype in cells.^2,9,38^ However, cellular morphological changes (hummingbird phenotype) were observed when the truncated construct was targeted to cellular membranes by adding a membrane-localization signal,^9^ demonstrating the importance of proper cellular localization, normally mediated by the SH2 domains. The relevance of association of SHP2 with its binding partners for its role in aberrant signaling has been demonstrated also by a study of monobodies targeting the N-SH2 domain and disrupting its association with adaptor proteins. Expression of these monobodies in cancer cells carrying the activating p.V45L substitution abolished ERK1/2 phosphorylation almost completely.^39^ Similarly, Kertész and co-workers^40^ reported that the natural SHP2 binding motif of Gab1, when delivered into immune cells, modulated phosphorylation patterns.

An example of the opposite situation, in which binding is preserved and the catalytic activity is impaired, is provided by *PTPN11* mutations causing NSML, such as p.T468M. This class of amino acid substitutions is located in the proximity of the PTP active site, at the PTP/N-SH2 interface, and have a twofold effect: they destabilize the closed state of the protein and consequently promote association of SHP2 with signaling partners; at the same time, they perturb the active site and therefore strongly impair the catalytic activity of the phosphatase. Interestingly, the phenotype of NSML is very similar to that of NS, and these mutations still allow the activation of multiple effector pathways.^22,41^

Overall, these findings strongly suggest that a mere enhancement of SHP2 catalytic activity is not sufficient to cause disease and indicate that an increased level of association with binding partners plays a major role in the pathogenic mechanism associated with *PTPN11* pathogenic variants. Therefore, inhibition of binding of SHP2 to other proteins through its SH2 domains represents a promising alternative pharmaceutical strategy. No molecules targeting the SH2 domains of SHP2 for therapeutic purposes have been developed so far, even though SH2 domains in general have received a great deal of attention as potential pharmaceutical targets.^42,43^ These recognition units generally have only moderate affinity and selectivity for cognate phosphorylated sequences, with dissociation constants in the range of 0.1–10 μM.^42,44−46^ However, we recently characterized the structural determinants of phosphopeptide binding by the N-SH2 domain of SHP2,^18^ and our data indicate this particular domain as a favorable exception, because its peculiar features make significantly higher affinities possible.

On the basis of these considerations, we explored the possibility of targeting SHP2 protein–protein interactions (PPIs), rather than its catalytic activity. We developed a peptide-based molecule with nanomolar affinity for the N-SH2 domain of SHP2, high specificity, and resistance to degradation. This inhibitor rescued the mortality and developmental defects induced by a pathogenic mutation *in vivo*. Our results provide a novel route for SHP2-targeted therapies and offer a new tool for further investigating the role of SHP2 PPIs in the signaling cascades controlled by this phosphatase.

## Results

### Characterization of IRS-1 pY1172/N-SH2 Binding

#### The IRS-1 pY1172 Peptide Binds the N-SH2 Domain with Nanomolar Affinity

The peptide corresponding to pY1172 (rat sequence, SLN-pY-IDLDLVKD) or pY1179 (human sequence, GLN-pY-IDLDLVKD) of insulin receptor substrate 1 (IRS-1) has one of the highest known binding affinities for the N-SH2 domain of SHP2.^18,47^ On the basis of our study of the structural determinants of a high affinity for this domain, the IRS-1 pY1172 sequence is near to optimal in several respects, because it has apolar residues at positions +1, +3, and +5, which point toward the hydrophobic groove in the N-SH2 structure, and anionic amino acids at positions +2 and +4, which can interact with a peculiar KxK motif in the BG loop.^18^

The binding affinity of the IRS-1 pY1172 peptide has been characterized in several literature studies. Unfortunately, these results are extremely contradictory, as reported in Table S1, with dissociation constants ranging from ∼10 nM to ∼10 μM. Several possible factors can be invoked to explain these discrepancies, including the effect of radioactive labels,^48^ dimerization of GST-N-SH2 constructs^49,50^ even at low nanomolar concentrations,^51^ or the sensitivity of the technique.^19^

**Figure 2:**
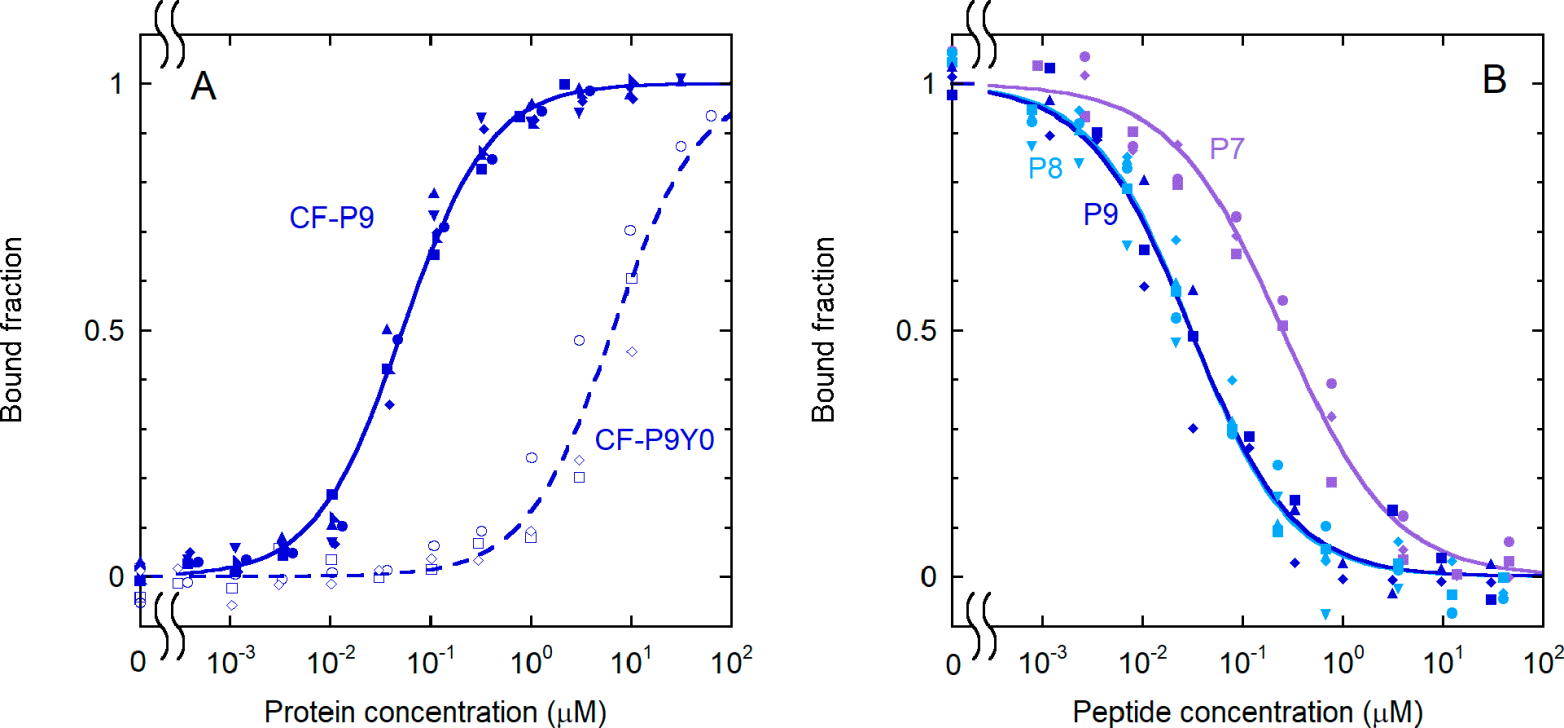
Effect of phosphorylation and sequence length on the binding of IRS-1 pY1172 peptides to the N-SH2 domain. (A) Binding curves for the phosphorylated and unphosphorylated sequence corresponding to the nine-residue region surrounding pY1172 of IRS-1 (see Table 1 for the sequences). The following experimental conditions were used: 1.0 nM CF-P9 (filled symbols and solid lines) and 10 nM CF-P9Y0 (empty symbols and dashed lines). (B) Displacement curves for unlabeled IRS-1 pY1172 analogues of different lengths [P9, P8, and P7 (see Table 1)]. A concentration of labeled peptide equal to 1.0 nM CF-P9, interacting with the N-SH2 domain (40 nM N-SH2), was displaced with increasing amounts of the unlabeled peptides. The bound fraction of labeled peptide is reported as a function of the concentration of the competing, unlabeled peptide. The results of independent, replicate experiments (*n* = 6 for CF-P9, *n* = 4 for P9 and P8, and *n* = 3 for CF-P9Y0 and P7) are reported with different symbols and were fit collectively.

Considering these difficulties, in this study, we developed a fluorescence anisotropy binding assay. In a direct binding experiment, the fluorescently labeled peptide IRS-1 pY1172 analogue CF-P9 (Table 1) was titrated with increasing concentrations of the N-SH2 domain. The fraction of protein-bound peptide was determined from the increase in fluorescence anisotropy (Figure 2), and a *K*_d_ of 53 ± 2 nM was obtained (Table 2).

**Table 1:**
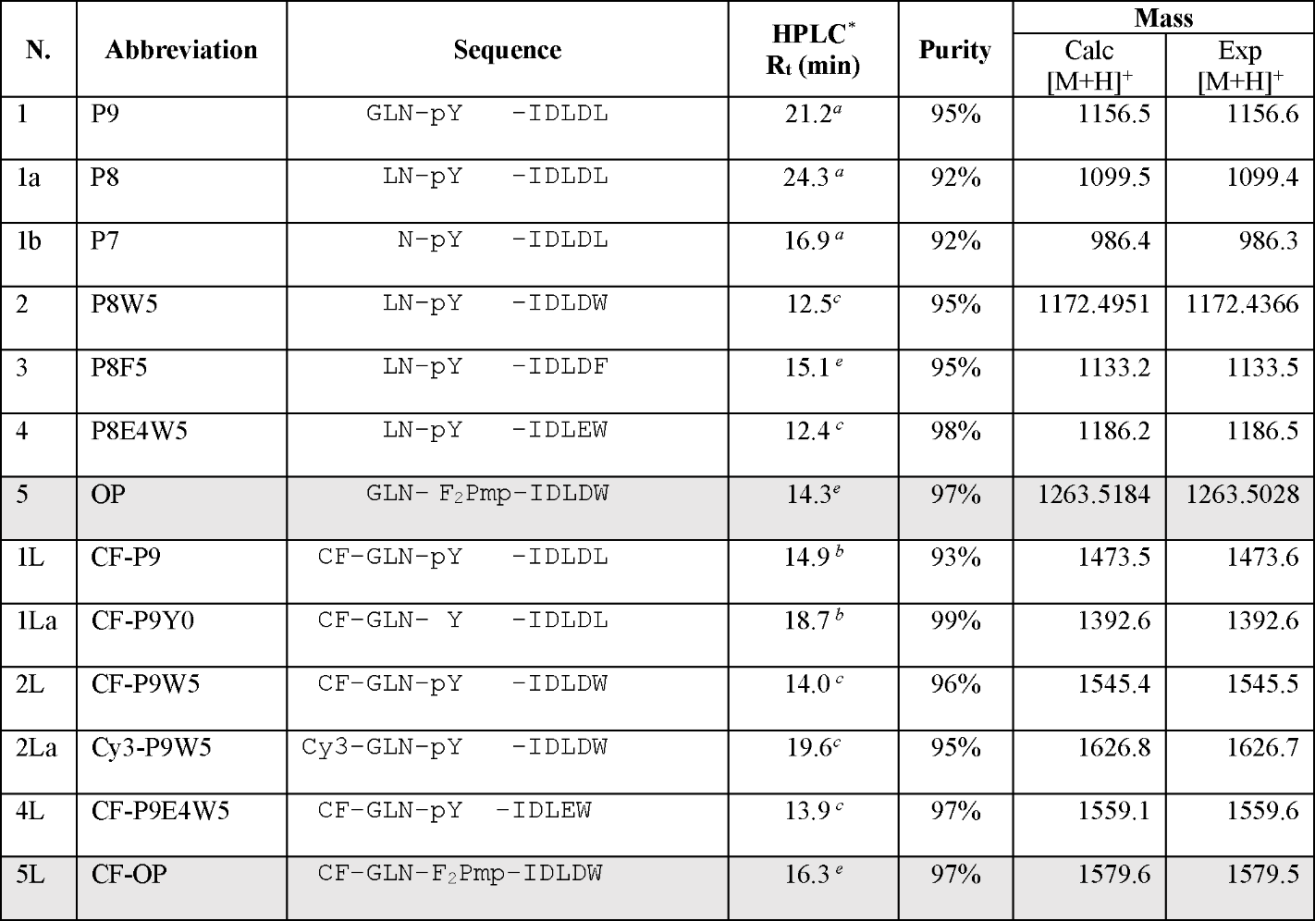
Peptide Sequences Investigated in This Study^a^

a All peptides were amidated at the C-terminus. Unlabeled peptides were acetylated at the N-terminus. CF is 5(6)-carboxyfluorescein, Cy3 is Cyanine 3 carboxylic acid, and F_2_Pmp is the nondephosphorylatable pY mimic phosphonodifluoromethyl phenylalanine. The optimized peptides are highlighted in gray. *RP-HPLC retention times (*R*_t_) and purities were determined with a Phenomenex Kinetex XB-C18 column (4.6 mm × 100 mm, 3.5 μm, 100 Å), with mobile phases A (aqueous 0.05% TFA) and B (acetonitrile, 0.05% TFA), with the following elution conditions: *^a^*10–40% B in 30 min, *^b^*20–50% B in 30 min, *^c^*20–60% B in 20 min, *^d^*10–95% B in 30 min, *^e^*5–95% B in 30 min, and *^f^*5–65% B in 30 min Theoretical molecular weights are compared with those determined experimentally by ESI-MS spectrometry or high-resolution ESI-Q-Tof (for the optimized peptide OP and its precursor P8W5).

**Table 2:**
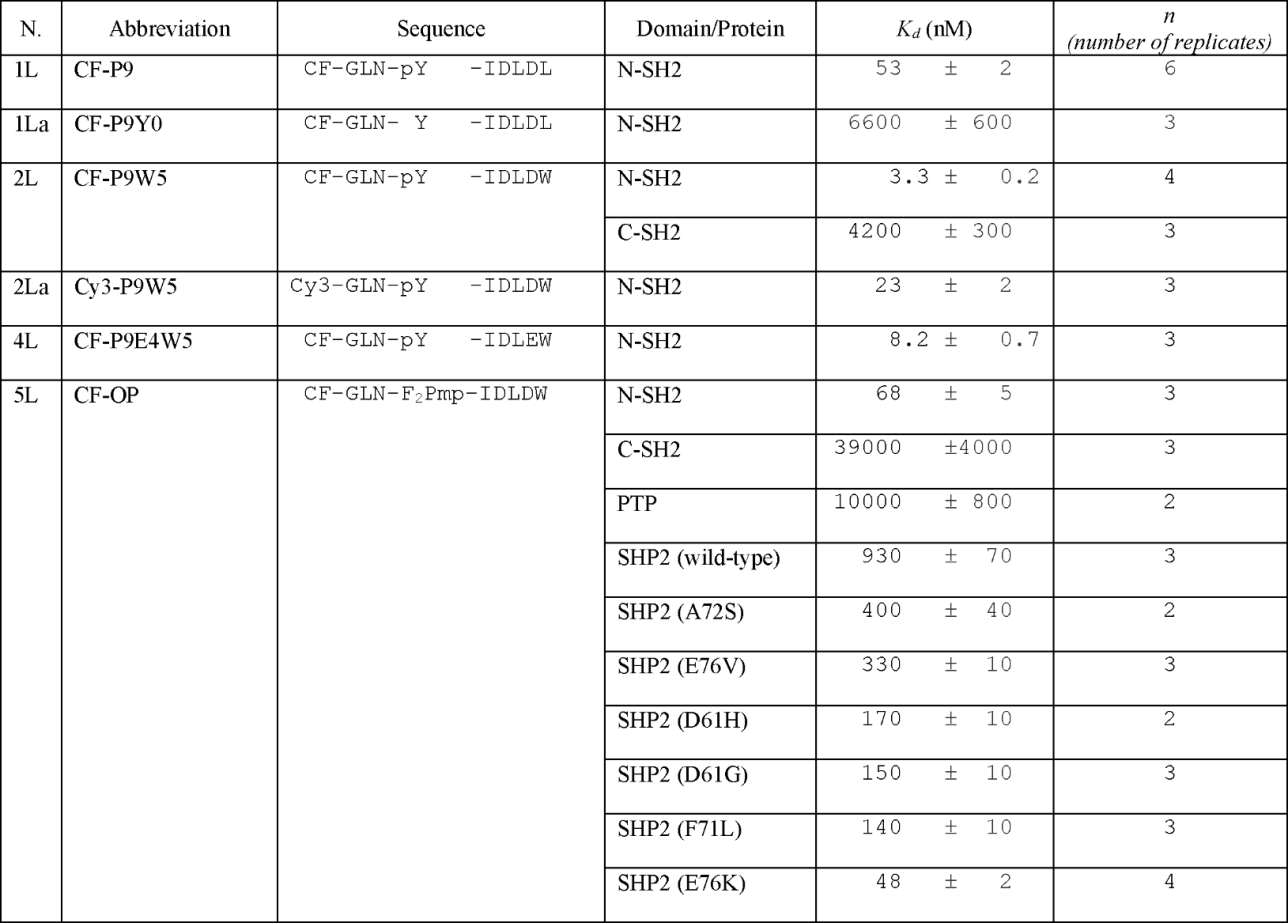
Dissociation Constants Obtained from the Fluorescence Anisotropy Binding Experiments^a^

a The dissociation constants and the associated errors are the results of collective fitting of independent, repeated experiments (as indicated in the number of replicates).

#### Phosphorylation Contributes Only 30% of the Standard Binding Free Energy

Association of SH2 domains with the partner proteins is regulated by phosphorylation, and therefore, the phosphate group is necessarily responsible for a large fraction of the binding affinity. At the same time, to have a good selectivity, the rest of the sequence must also contribute significantly to the peptide/protein interaction. To quantify this aspect, we performed a binding experiment (Figure 2A) with an unphosphorylated analogue of the labeled IRS-1 pY1172 peptide, CF-P9Y0 (Table 1). The affinity was approximately 100 times lower, with a *K*_d_ of 6.6 ± 0.6 μM, compared with a *K*_d_ of 53 ± 2 nM for the phosphorylated peptide. The corresponding values for the standard free energy of binding (assuming a 1 M standard state) are −29.6 ± 0.2 and −41.6 ± 0.1 kJ/mol, respectively. Assuming additivity of contributions, the phosphate group is responsible for the difference of −12.0 ± 0.2 kJ/mol, i.e., for <30% of the total standard binding free energy of the phosphorylated peptide. This result indicates that the contribution of the rest of the peptide predominates in the binding interactions and bodes well for our design efforts.

### Sequence Optimization

#### The Phosphopeptide Sequence Can Be Reduced to Eight Amino Acids without a Loss of Affinity

Literature data are partially contradictory regarding the effect of shortening the IRS-1 pY1172 sequence on the binding affinity. Kay^52^ reported that the sequence could be shortened at the C-terminus down to residue +5 and at the N-terminus down to residue −2, without any loss of affinity. By contrast, Case^48^ observed a significant decrease in affinity by shortening the sequence from SLN-pY-IDLDLVKD to LN-pY-IDLDLV. Our previous study clearly indicated that residues −2 to +5 are the most important for the interaction.^18^ To clarify the role of N-terminal residues in determining the N-SH2 domain binding affinity, we performed displacement studies (Figure 2B) with the unlabeled peptide P9 and with the shortened analogues P8 and P7 (Table 1), where residues −3 or −2 and −3 were removed, respectively. No significant loss of affinity was observed by reducing the sequence to eight residues, while removal of the amino acid at position −2 caused a drastic perturbation of the complex stability (Figure 2B). The −2 to 5 IRS-1 sequence is the minimal peptide with a nanomolar dissociation constant.

#### Single-Amino Acid Substitutions in the IRS-1 pY1172 Sequence Improve the *K*_d_ to the Low Nanomolar Range

Hydrophobic residues are required at positions +1, +3, and +5 of the phosphopeptide sequence,^18^ but aromatic residues are present in some natural high-affinity binding sequences, at position +5 only.^8,10,48,53,54^ The crystallographic structures of some of these complexes^8,10,24^ show that an aromatic side chain can be accommodated by a relatively large hydrophobic pocket and that the peptide residue 5 interacts with the BG and EF loops of the domain, which are important for binding specificity.^18,24^ Finally, a preference for aromatic residues at position +5 has been indicated by several peptide library studies.^36,55−57^

**Figure 3:**
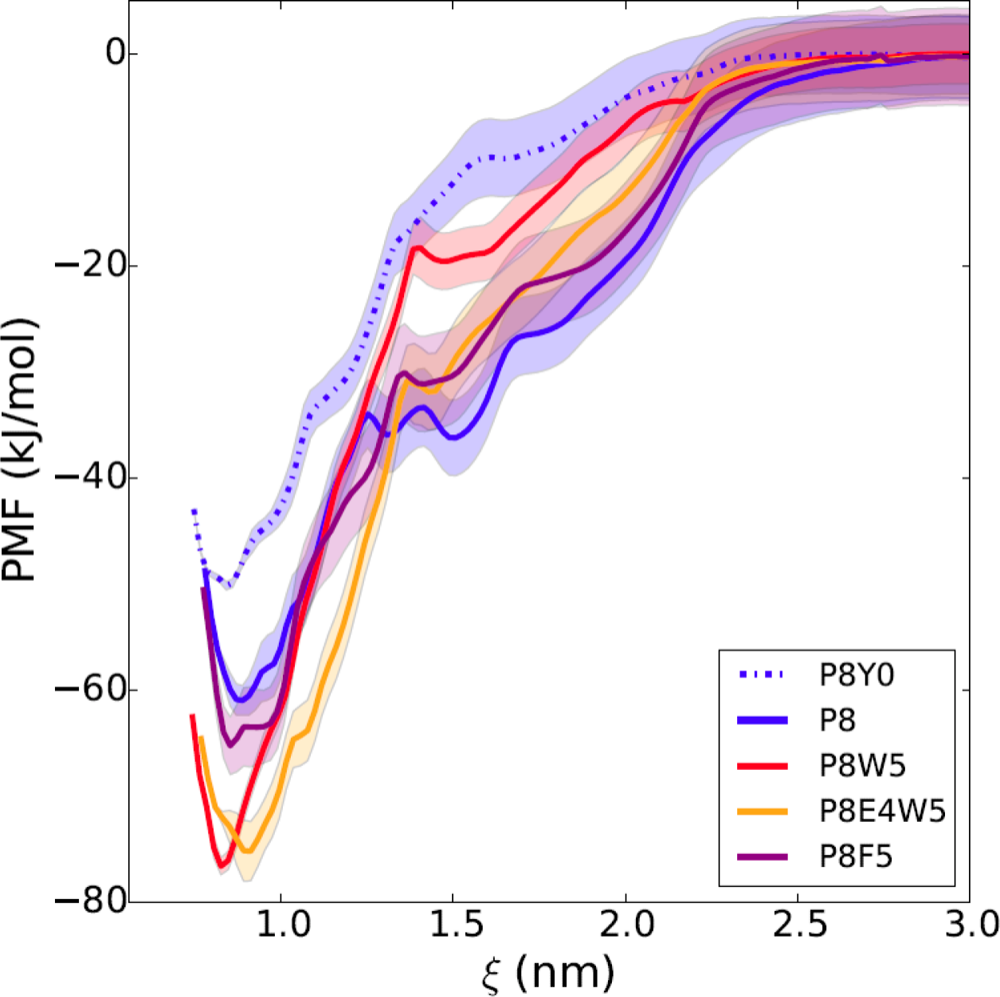
*In silico* free energy calculations for different modified sequences. The free energy profile is reported as a function of the distance between the centers of mass of the N-SH2 domain and the phosphopeptide. The simulations predict a loss of affinity of P8 (blue line) with dephosphorylation of the pY (dashed blue line; P8Y0 is the unphosphorylated analogue of P8) and a gain with substitution of L at position +5 with W (red line), but not with F (violet line). The additional substitution of D at position +4 with E (orange) does not provide any further increase in affinity. Shaded areas correspond to standard deviations in the PMF profile. For peptide sequences, see Table 1.

On the basis of these considerations, we predicted *in silico* the effect of different aromatic amino acids at position +5. Free energy calculations indicated that substitution of L with the bulkier W (but not with F) could be favorable (Figure 3). The additional substitution of D at position +4 with the longer E was evaluated, as well, fora possible strengthening of the electrostatic interactions with the KxK motif in the BG loop. However, in this case no further increase in binding affinity was predicted by the free energy calculations (Figure 3).

**Figure 4:**
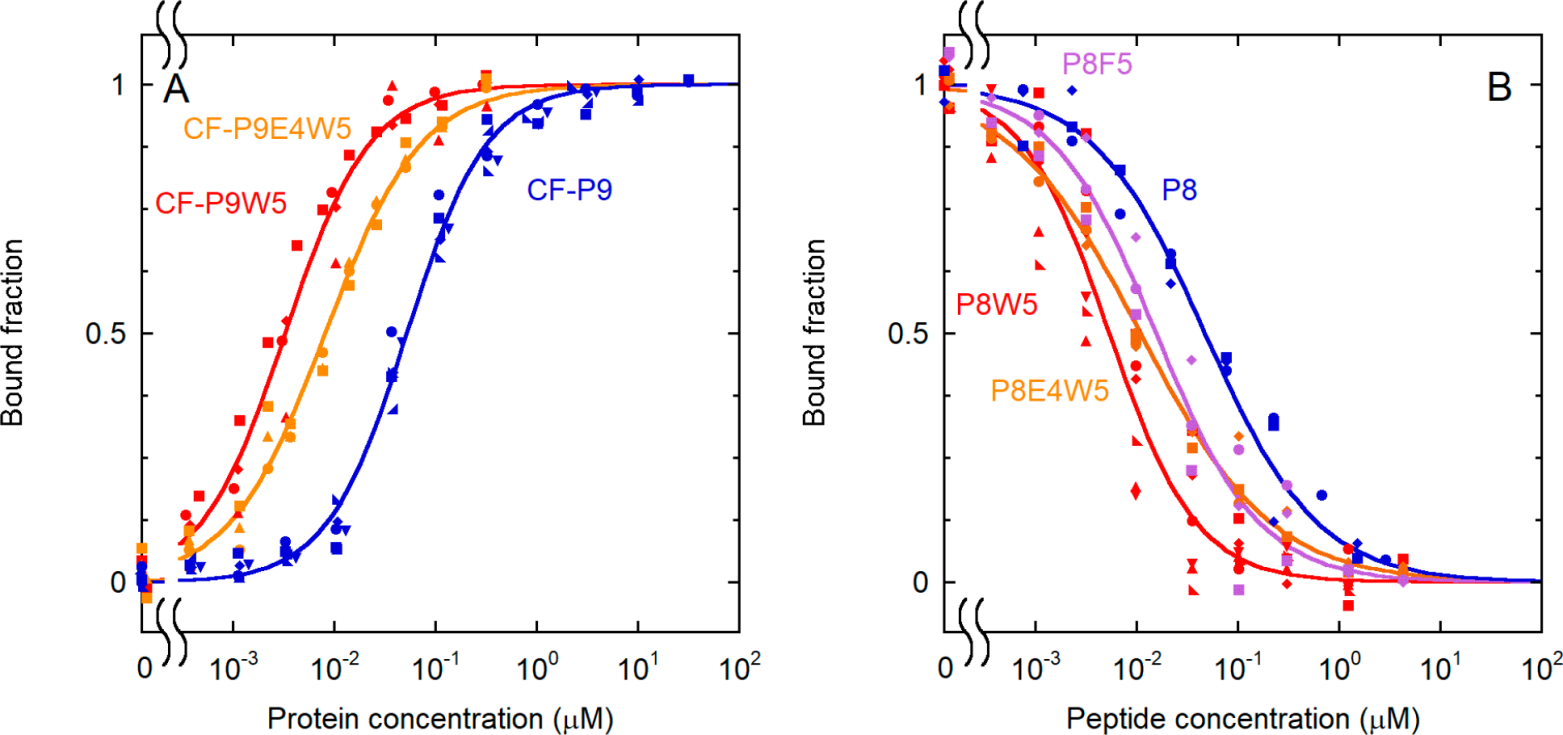
Effect of substitutions at position +5 on binding affinity. (A) Direct binding experiments with various analogues (for the peptide sequences, see Table 1). Substitution of L5 with W caused a dramatic increase in binding affinity, which was partially lost with the additional substitution of D4 with E. The following experimental conditions were used: 0.10 nM CF-P9W5, 0.10 nM CF-P9E4W5, and 1.0 nM CF-P9. Data for CF-P9 are repeated here for comparison. (B) Displacement assay, performed with various analogues (for the peptide sequences, see Table 1). A concentration of labeled peptide equal to 0.10 nM CF-P9W5, interacting with the N-SH2 domain (3.3 nM N-SH2), was displaced with increasing amounts of the unlabeled peptides. The bound fraction of the labeled peptide is reported as a function of the concentration of the competing, unlabeled peptide. The results of independent, replicate experiments (*n* = 5 for CF-P9 and P8W5, *n* = 4 for CF-P9W5, and *n* = 3 for CF-P9E4W5, P8, P8F5, and P8E4W5) are reported with different symbols and were fit collectively.

Analogues with F or W at position +5 (P8F5 or P8W5, respectively), as well as a labeled analogue with the L to W substitution (CF-P9W5) (Table 1), were synthesized and studied experimentally (Figure 4). As predicted, introduction of W at position +5 was highly favorable, leading to a decrease in the dissociation constant by one order of magnitude, for the labeled and unlabeled analogue (Tables 2 and 3). Consequently, the dissociation constant for the P8W5 analogue was 1.6 ± 0.2 nM. By contrast, the additional D to E substitution resulted in a slight loss of binding affinity (Figure 4 and Tables 2 and 3).

**Table 3:**
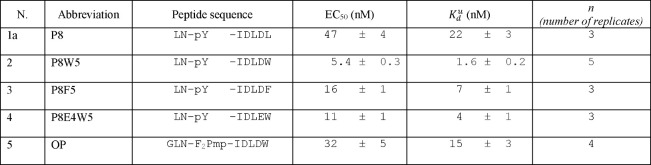
Dissociation Constants Obtained from Displacement Experiments^a^

a All measurements were performed on the N-SH2 domain of SHP2. Experiments were performed at 3.3 nM N-SH2 and 0.5 nM CF-P9W5 (for P8 and OP) or 0.1 nM CF-P9W5 (for the other peptides). EC_50_ indicates the concentration needed to displace half of the labeled peptides, while *K*_d_^u^ is the dissociation constant of the unlabeled peptide (see the Supporting Information). EC_50_ values and the associated errors are the results of collective fitting of independent, repeated experiments (as indicated in the number of replicates).

On the basis of these results, further studies concentrating on the peptide with W at position +5 were conducted.

### Binding Selectivity

#### The Modified Sequence Is Highly Selective for the N-SH2 Domain of SHP2

The selectivity of binding of CF-P9W5 was first assessed with respect to the C-SH2 domain of SHP2 by fluorescence anisotropy (Figure S1). As reported in Table 2, the affinity for the C-SH2 domain was almost 1000 times lower than that for the N-SH2 domain.

**Figure 5:**
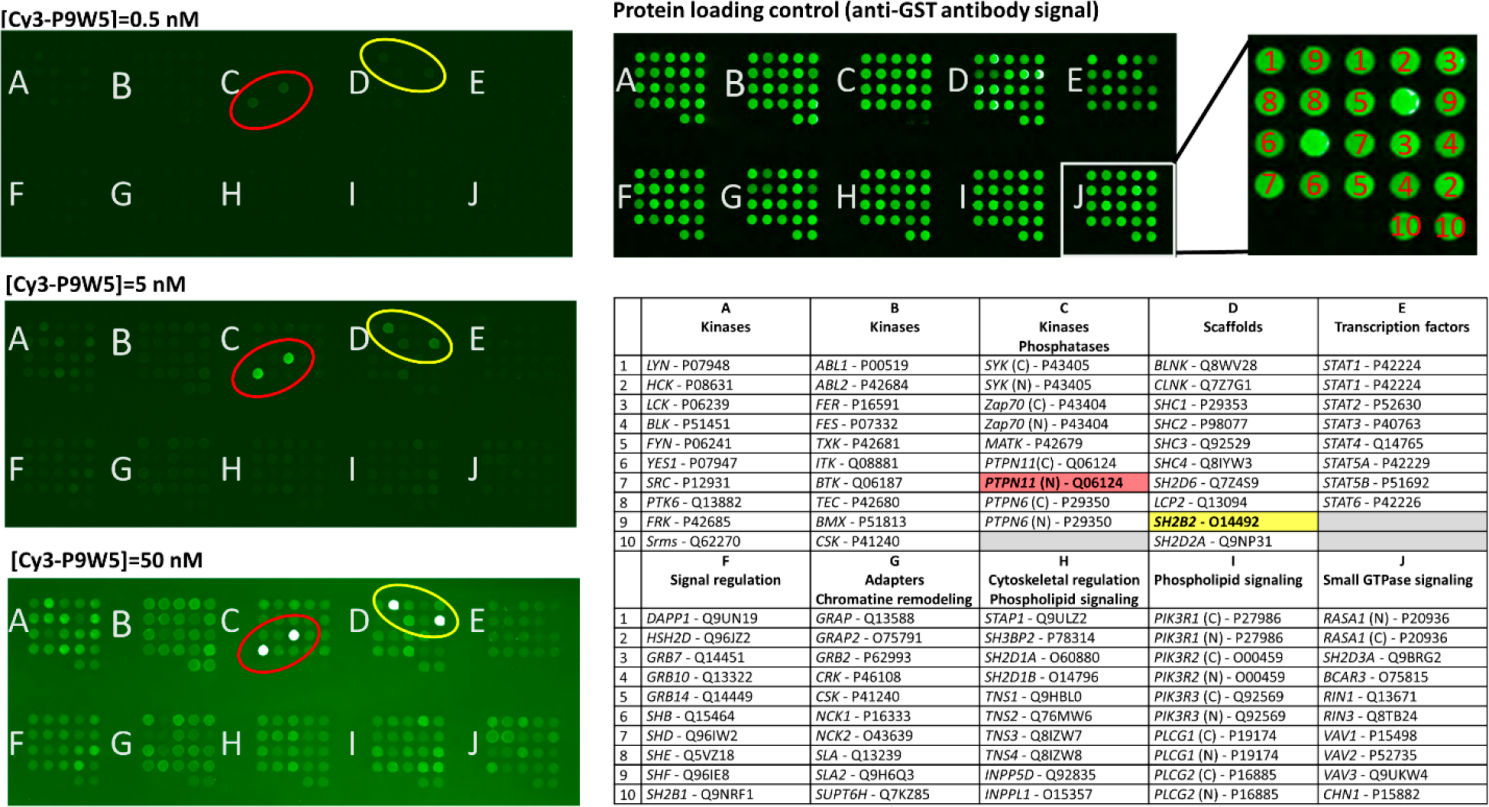
Binding selectivity of Cy3-P9W5 for an array of SH2 domains. In the left panels, the fluorescently labeled analogue Cy3-P9W5 was allowed to interact with an array of human SH2 domains, expressed and purified as glutathione *S*-transferase (GST) fusion constructs. Binding affinity was assessed by the fluorescence of the bound peptide, at concentrations of 0.5 nM (top), 5.0 nM (center), and 50 nM (bottom). Each SH2 domain was spotted in duplicate, and negative control spots (with GST only) are also present. The bright spots correspond to the N-SH2 domain of SHP2 (circled in red) and to the SH2 domain of the SH2 and PH domain-containing adapter protein APS (also called SHP2B2, circled in yellow). The intensity of all other spots is comparable to that of the negative controls. The right panels shows a control of the protein loading in each spot, performed with an anti-GST antibody (top), and the position of each SH2 domain in the array (bottom). For each SH2 domain, the gene name and Uniprot codes are reported. Each domain was spotted in duplicate.

A more complete analysis of the binding selectivity was performed on a protein array considering 97 human SH2 domains (Figure 5). An analogue of CF-P9W5 was employed in this assay, where CF was substituted with the Cy3 dye, suitable for detection in the array reader. Control binding experiments showed that the change in fluorophore affected peptide binding affinity only marginally (Table 2 and Figure S2). Strikingly, significant binding was observed only with the N-SH2 domain of SHP2 and, to a lesser extent, the SH2 domain of the adapter protein APS (also called SHP2B2). It is worth noting that binding to the N-SH2 domain of SHP1, which is the most identical to SHP2,^17^ was negligible.

### Engineering Resistance to Degradation

#### Introduction of a Nonhydrolyzable pY Mimic Is Compatible with Nanomolar Binding Affinity

For intracellular or *in vivo* applications of the phosphopeptide, it is essential to make it resistant to degradation. The most labile moiety is the phosphate group of the pY residue, which can be hydrolyzed by protein tyrosine phosphatase, possibly also including SHP2, of which IRS-1 pY1172 has been shown to be a substrate.^58^ We substituted the pY with the nonhydrolyzable mimetic phosphonodifluoromethyl phenylalanine (F_2_Pmp), which is isosteric with pY and has a total negative charge comparable to that of pY under physiologic pH conditions.^43,59^

Binding experiments demonstrated that the substituted analogue [CF-OP, for the optimized peptide (Table 1)] has a dissociation constant for the N-SH2 domain that is just one order of magnitude worse with respect to that of CF-P9W5 (68 ± 5 nM vs 3.3 ± 0.2 nM) (Figure S3 and Table 2). Similarly, the dissociation constant for the unlabeled peptide OP was 15 ± 3 nM (1.6 ± 0.2 nM for P8W5) and thus remained in the nanomolar range (Table 3). Binding experiments with the C-SH2 domain confirmed that the optimized peptide maintained the selectivity of the parent sequence (Figure S3 and Table 3).

#### The Optimized Peptide OP Is Resistant to Proteolytic Degradation

To test resistance to proteases, the optimized peptide OP and P8W5 were incubated in human serum for 24 h and then analyzed by HPLC. Both peptides were degraded by <50% after 1 day. By comparison, the decapeptide XP (WFKYYGKAIY, with free termini),^60^ which we used as a positive control, was totally degraded in 3 h (Figure S4). Similarly, OP was stable in DMEM for 3 days (data not shown). These results are probably due to the presence of the pY or F_2_Pmp residues and to the protected termini, and they bode well for potential *in vivo* applications of the peptides.

### Binding to and Activation of the SHP2 Protein

#### Pathogenic Mutants Bind to Phosphopeptides with an Affinity Much Higher Than That of the Wild-Type Protein

As discussed in the Introduction, we and others have hypothesized that, in its autoinhibited state, the conformation of the N-SH2 domain prevents efficient association with binding partners, while the affinity of SHP2 for phosphorylated sequences is maximized in the open, active state.^19,21−23^ This model has many relevant consequences, because it implies that pathogenic mutants have a twofold effect: they increase the activity of the phosphatase but also its affinity for binding partners. In principle, both effects could be the origin of the hyperactivation of the signal transduction pathways involved in the pathologies caused by *PTPN11* mutations.

**Figure 6:**
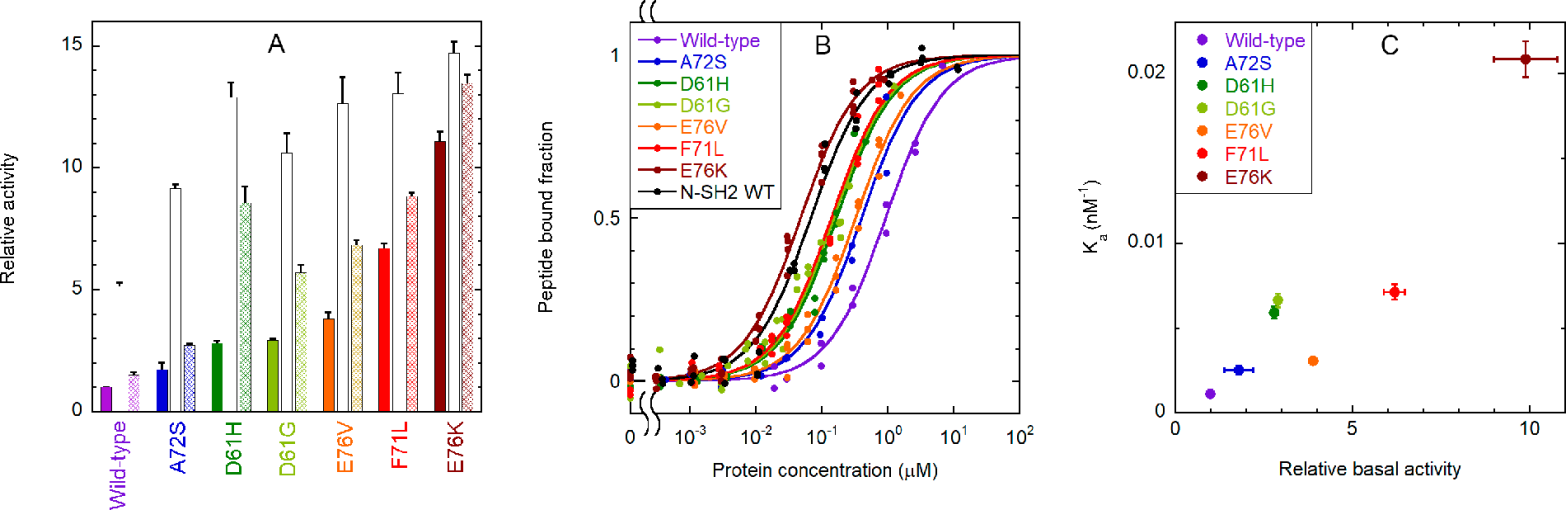
Binding of the CF-OP peptide to the whole SHP2 protein (wild type and pathogenic mutants) and activation of the phosphatase activity. (A) Relative catalytic activity of the wild-type protein and selected pathogenic mutants, under basal conditions (filled bars) and after stimulation with 10 μM BTAM peptide (empty bars) or 10 μM OP (dashed bars). All values were normalized to the basal activity of the wild-type protein. Each experiment was performed in triplicate. Binding to phosphorylated sequences induces activation. All mutations cause an increase in basal activity by destabilizing the autoinhibited conformation, and a concomitant enhanced responsiveness to activating phosphopeptides. (B) Curves for binding of the CF-OP peptide to the wild-type protein and selected mutants, obtained from fluorescence anisotropy experiments (1.0 nM CF-OP). Independent replicate experiments (*n* = 4 for E76K, *n* = 3 for E76V, F71L, D61G, and wild-type N-SH2, and *n* = 2 for the wild type, A72S, D61H) were fitted collectively. (C) Correlation between the relative basal activity of the various mutants (as reported in panel A) and their binding affinity (association constant, i.e., 1/*K*_d_) for CF-OP. An enhanced basal activation of the protein, caused by destabilization of the autoinhibited state, is accompanied by an increased affinity for the CF-OP peptide. Therefore, CF-OP binds more tightly to the most activating mutants, which also cause the strongest pathogenic effects. Error bars represent standard deviations.

Notwithstanding the relevance of this aspect, to the best of our knowledge, no direct phosphopeptide binding experiments with whole SHP2 protein have ever been performed, possibly due to the fact that pY can be dephosphorylated by the PTP domain (see the next section). Now, OP and its fluorescent analogue CF-OP allow us to directly assess the hypothesis described above. Figure 6 and Table 2 report the results of binding experiments performed with CF-OP and wild-type SHP2 or the pathogenic mutants A72S, E76K, D61H, D61G, F71L, and E76V. E76K is among the most common somatic lesions associated with leukemia and has never been observed as a germline event in individuals with NS,^3,20^ as it results in early embryonic lethality.^61^ This variant is strongly activating, with the basal activity of the corresponding mutant being at least 10 times higher than that of the wild-type protein. Conversely, A72S is a germline mutation specifically recurring among subjects with NS. In this case, basal activation is only twofold.^21^ In humans, the D61G substitution has been found in both NS and leukemia,^62^ and in animal models, it induces both NS-like features and myeloproliferative disease.^63^ The D61H, F71L, and E76V amino acid substitutions have been identified as somatic events in JMML and other leukemias,^3^ and when transmitted in the germline, they are associated with a high prenatal lethality (M. Zenker, personal communication, September 2019). Interestingly, we observed that the affinity for CF-OP nicely parallels the basal activity of these mutants (Figure 6). This finding provides a first direct confirmation that the the affinity of SHP2 for phosphorylated binding partners is higher in the open, active conformation than in the closed, autoinhibited state.

#### OP Is Also an Inhibitor of the PTP Domain

On the basis of previous reports of the dephosphorylation of IRS-1 pY1172 by SHP2,^58^ we verified if P8 and P8W5 are also substrates of this protein. These experiments were performed with a truncated SHP2 construct lacking the N-SH2 domain (SHP2_Δ104_), as it is fully activated and was shown to be more stable and less prone to aggregation than the isolated PTP domain.^37^ As reported in Figure S5, dephosphorylation was indeed observed, although to a lesser extent than for other phosphopeptides. Using the nondephosphorylatable peptide CF-OP, we measured directly binding to the PTP domain of SHP2 (Figure S6 and Table 2). Significant association was observed, although with a much lower affinity than with the N-SH2 domain (*K*_d_ = 10.0 ± 0.8 μM).

These findings indicate that, in principle, the nondephosphorylatable OP could act as a double-hit SHP2 inhibitor, acting on both PPIs and catalytic activity. In addition, they underline the importance of substituting pY with a nondephosphorylatable mimic: in principle, any high-affinity phosphopeptide ligand of the N-SH2 domain (including natural sequences) could be used to inhibit association of SHP2 with its binding partners, but these sequences could be dephosphorylated by SHP2 itself, losing their binding affinity.

#### OP Activates SHP2 Only Weakly

Binding of mono- or bisphosphorylated peptides causes activation of SHP2. We tested the effect of OP on the wild-type and mutated proteins. As shown in Figure 6, activation was generally weak, compared with that induced by the bisphosphorylated BTAM peptide. Interestingly, under the experimental conditions used (10 μM peptide), the N-SH2 domain of the wild-type and mutant proteins is nearly saturated by the OP, according to the binding experiments reported in Figure 6. This finding indicates that the peptide could inhibit SHP2 PPIs, causing only a limited increase in catalytic activity. In any case, as demonstrated by studies of truncated constructs lacking the N-SH2 domain,^2,9,38^ activation of SHP2 without proper PPIs has no pathogenic effects.

### OP Reverses the Effects of D61G Mutation *In Vivo*

We used the zebrafish model system to explore the *in vivo* effect of the peptide. Zebrafish Shp2a is highly homologous to human SHP2 (91.2% protein sequence identity); in particular, the sequences of the N-SH2 domain and the N-SH2/PTP interface are identical in the human and fish proteins. RASopathy-associated mutants, including activating mutants of Shp2a, greatly impact zebrafish development. Microinjection of synthetic mRNA encoding NS-associated mutants of Shp2 at the one-cell stage induces NS-like traits.^64^ During gastrulation, convergence and extension movement are affected, resulting in oval-shaped embryos, with an increased major/minor axis length ratio 11 h postfertilization (hpf).^64^ Here we co-injected Shp2a-D61G mRNA with OP in zebrafish embryos to investigate whether OP rescues the defective cell movements during gastrulation.

**Figure 7:**
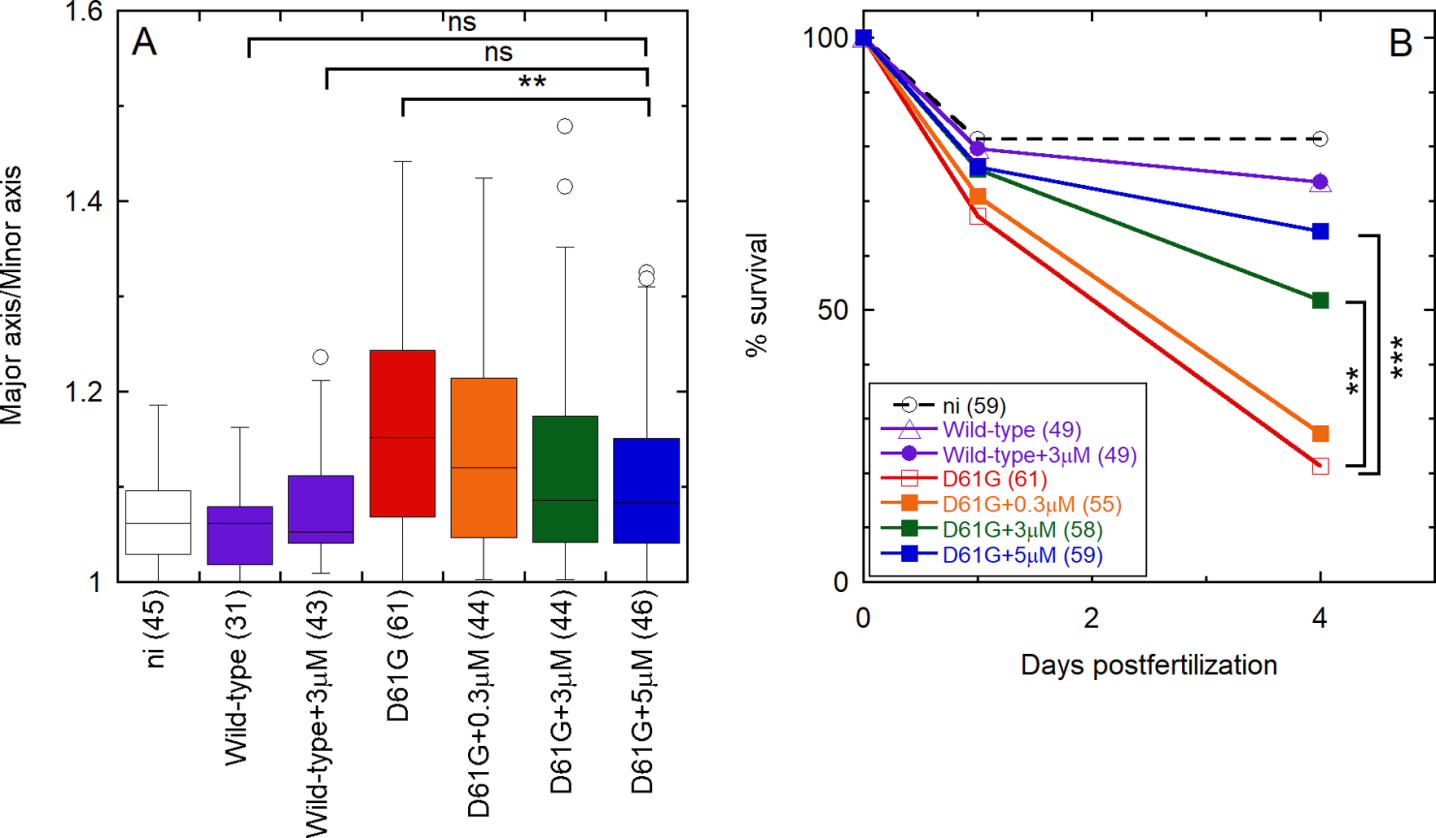
OP partially rescues D61G Shp2a-induced gastrulation defects and mortality in a dose-dependent manner in zebrafish embryos. Embryos were injected at the one-cell stage with mRNA encoding GFP-2A-Shp2-D61G or GFP-Shp2-wild-type with or without the peptide at concentrations of 0.3, 3, and 5 μM. Non-injected embryos (ni) were evaluated as a control. (A) Ovality of embryos 11 hpf, as indicated by the ratio of the long and short axis. Tukey’s honest significant difference test was performed to assess significance. In the box plot, the horizontal line indicates the median, box limits indicate the 25^th^ and 75^th^ percentiles (interquartile range), and whiskers (error bars) extend to the maximum and minimum values, or to 1.5 times the interquartile range from the 25^th^ and 75^th^ percentiles, if some data points fall outside this range. In the latter case, outliers are indicated as single data points. (B) Embryo lethality. Surviving embryos were counted 1 and 4 days post fertilization. Survival was plotted, and a log rank test was performed to access differences between groups. Nonsignificant (n.s.), *p* > 0.05; *p < 0.05; **p < 0.01; ***p < 0.001. The numbers of embryos that were analyzed in each case are indicated in parentheses, in the axis labels (A), or in the legend (B) and ranged from 31 to 61.

As shown in Figure 7 and Figure S7, we observed a dose-dependent decrease in Shp2a-D61G-induced major/minor axis ratios, with a rescue of the phenotype that was significant at a peptide concentration of 5 μM. On the contrary, embryos injected with wild-type Shp2a were almost perfect spheres at 11 hpf, and co-injection with 3 μM peptide had no impact on their shape. As expected, a large portion of D61G Shp2a-injected embryos were severely affected and died during embryonic development, whereas injection of wild-type Shp2a did not induce significant lethality. We followed the survival of D61G Shp2a-injected embryos and observed a significant and dose-dependent improvement in the survival of embryos upon co-injection with 0.3, 3, and 5 μM OP (Figure 7B). By contrast, the lethality of wild-type Shp2a embryos was not affected by co-injection of 3 μM OP. Altogether, these results indicate that co-injection of the OP rescued the developmental defects induced by a pathogenic, basally activated Shp2a variant, while it had no effect on wild-type embryos.

## Discussion

Here, we developed peptide-based molecules with nanomolar affinity for the N-SH2 of SHP2, good selectivity, stability to degradation, and an affinity for pathogenic variants of SHP2 up to 20 times higher than for the wild-type protein, as a novel strategy for targeting upregulated SHP2 function. Our findings also provide several insights into the interaction of phosphopeptides with SH2 domains and, in particular, with the N-SH2 domain of SHP2 and into the suitability of these recognition units as therapeutic targets.

Soon after their discovery, the affinities of SH2 domains for their binding partners (i.e., the dissociation constants) were considered to fall in the range of 10–100 nM.^67^ However, it turned out that most of the binding studies performed in that period were affected by experimental artifacts, leading to an overestimation of the binding affinities.^44,50^ A reassessment of the affinity values led to a commonly accepted range on the order of 0.1–10 μM.^42,44−46^ Such moderate affinities are considered to be crucial for allowing transient association and dissociation events in cell signaling. Consistently, SH2 domains artificially engineered to reach low nanomolar affinities for phosphorylated sequences (known as superbinders),^68^ by increasing the affinity of the domain for the pY residue, have detrimental consequences for signal transduction. However, micromolar binding affinities make SH2 domains in general less than optimal therapeutic targets.

In the case of the N-SH2 domain of SHP2, literature results on the affinity for the IRS-1 pY1172 peptide were contradictory, with dissociation constants varying by 3 orders of magnitude.^19,48,49^ Here, we showed that, at least for this peptide, the dissociation constant is in the nanomolar range. For the N-SH2 domain, similar affinities have been reported also for GRB2-associated binding protein 1 (Gab1), pY627,^69^ and Gab2, pY614,^54^ and several other peptides have dissociation constants within one order of magnitude of that of IRS-1 pY1172.^8,17^ In addition, in the study presented here, we were able to further improve the affinity with respect to the parent peptide. Therefore, the N-SH2 domain of SHP2 might constitute an exception in the panorama of SH2 domains, regarding binding affinity. In most cases, interaction of phosphopeptides with SH2 domains is dominated by the hydrophobic effect (except for the pY pocket). However, the N-SH2 domain of SHP2 has a peculiar KxK motif in the region of the BG loop pointing toward the binding groove, which can interact electrostatically with acidic residues present in the peptide sequence at positions +2 and +4.^18^ Therefore, by contrast to the superbinders, the high binding affinity of the N-SH2 domain is a result of additional interactions in the selectivity-determining region, and not in the pY pocket. Indeed, our data showed that the pY phosphate contributed <30% of the standard binding free energy. This finding is comparable to what has been reported for other SH2 domains.^70^

Our results also showed that residue −2 contributes significantly to the binding affinity. Indeed, while the specificity of most SH2 domains is determined by residues C-terminal to the pY, peptide library and array studies have shown that, contrary to most other SH2 domains, the N-SH2 domain of SHP2 has specific preferences for position −2.^18^ This peculiarity is due to the fact that, in place of the commonly conserved arginine at position 2 in the first α-helix (αA2), the N-SH2 domain of SHP2 has G13. Consequently, a hydrophobic peptide residue at position −2 can insert in the space left free by the missing protein side chain and interact with V14 αA3 and with the phenol ring of pY, stabilizing its orientation and the overall complex.^18^

A preference of the N-SH2 domain of SHP2 for hydrophobic residues at positions +1, +3, and +5 is well-established. These side chains insert into the groove on the surface of the domain and interact with exposed hydrophobic patches.^18^ Now our data demonstrate that the bulky, aromatic side chain of tryptophan at position +5 is 10 times better (in terms of dissociation constant) than the leucine residue, which is present in high-affinity natural sequences such as those of IRS-1, Gab1, and Gab2. Overall, these data confirm that the phosphopeptide sequence in the −2 to +5 stretch contributes significantly to the binding affinity. In principle, highly specific binding should be possible.

In general, SH2 domains are only moderately discriminating for binding target sequences, and a range of residues is tolerated at each site.^44,70^ Consequently, nonspecific tyrosine-phosphorylated sequences are usually bound only 10–100-fold more weakly than specific targets.^42,45,70,71^ Indeed, additional specificity is often provided by tandem SH2 domains:^70^ two closely spaced tyrosine-phosphorylated motifs bind to tandem SH2 domains with 20–50-fold greater affinity and specificity compared with the binding of a single SH2 domain with a single tyrosine-phosphorylated motif.^45^ SHP2 and its SH2 domains are no exception in this case, as peptide library and array studies, together with the sequences of known natural binding partners, showed a significant variability in the sequences of peptides bound by SHP2.^18^ However, our results indicate that some peptides (like those developed here) can bind specifically to a single SH2 domain. Among an array of 97 human SH2 domains, we found some interference only with adapter protein APS (also called SHP2B2). The structure of the APS SH2 domain in complex with a cognate protein shows that the phosphorylated sequence binds in a folded, kinked conformation, rather than in the usual extended binding mode.^72^ This observation should facilitate the further development of our peptides, to avoid the unwanted interaction with APS, without affecting the affinity for the target N-SH2.

Finally, it is worth mentioning that approximately one order of magnitude in affinity was lost by substituting the pY residue with the nondephosphorylatable mimic F_2_Pmp. This finding is consistent with previous studies showing that F_2_Pmp is tolerated differently by various SH2 domains, and its insertion in place of pY in a phosphopeptide sequence can lead either to a loss or to an increase in affinity, by approximately one order of magnitude.^59^ Further optimization of this aspect is warranted, but the dissociation constant of our nondephosphorylatable peptide remained in the nanomolar range.

The nondephosphorylatable peptide allowed novel experiments on several aspects of SHP2 function and regulation, previously complicated by the fact that many phosphorylated sequences that bind to the N-SH2 domain are also substrates of the PTP catalytic site. Examples include IRS-1,^49,58^ Gab1,^73,74^ Gab2,^75^ PDGFR,^76,77^ PD-1,^8^ and SHPS-1.^78,79^ The OP, developed here, avoids the practical difficulty of dephosphorylation by SHP2 itself during the experiments.

As discussed in the Introduction, in the autoinhibited state of SHP2, the N-SH2 binding groove is closed, apparently making phosphopeptide association impossible. By contrast, the N-SH2 binding site is open in the structure of the isolated N-SH2 domain or of active SHP2. Consequently, it has been hypothesized that mutations destabilizing the closed state and favoring SHP2 activation could lead to an increase in binding affinity.^21^ This idea is indirectly supported by the fact that basally activated mutants require lower concentrations of SH2 domain binding phosphopeptides to reach full activation.^19,21−23^ However, no direct measurements of the binding of the phosphopeptide to different SHP2 variants had been reported until now. Our data directly demonstrate that the affinity for phosphopeptides of activated variants of SHP2 can increase by a factor of 20, reaching the same value as that of the isolated domain in the most active mutants. This consequence of pathogenic mutations adds to the increase in basal activity and might be the main factor responsible for hyperactivation of signaling pathways modulated by SHP2. Interestingly, for possible therapeutic applications, in a cellular environment, N-SH2 targeting peptides, such as those developed here, would act as effective inhibitors of the PPIs of mutant, hyperactivated SHP2, while they would have a much weaker effect on the wild-type protein. This behavior is the exact opposite of what has been observed for allosteric inhibitors, such as SHP099, which have significantly impaired activity in pathogenic variants of SHP2.^23,25^

A second link between SHP2 activity and binding functions is provided by the fact that SHP2 interactors are both ligands of the SH2 domains and substrates of the catalytic PTP domain, often with the same phosphorylated sequence. We showed here that our modified sequence can be dephosphorylated, too, when it comprises a normal pY residue. These data indicate the possible presence of a still uncharacterized feedback mechanism for regulating SHP2 signaling. Using our nondephosphorylatable peptide, we could demonstrate that a N-SH2 binding sequence associates with the catalytic PTP domain, too, although with a lower affinity. This finding suggests that it might be possible to develop double-edged sword molecules, which can inhibit both the catalytic activity and the PPIs of SHP2.

Phosphorylated sequences cause SHP2 activation by binding to the N-SH2 domain and inducing or stabilizing a domain conformation that is incompatible with the N-SH2/PTP interaction. In principle, it is possible that different phosphopeptide sequences do not have the same aptitude for causing or favoring the conformational transition of the N-SH2 domain needed for SHP2 activation. In this case, the binding affinity and activating potential of phosphopeptides would not be strictly coupled. Some literature data indicate that this might be the case. For instance, the sequences corresponding to pY546, pY895, and pY1222 of IRS-1 (rat ortholog numbering)^48,49^ or artificial sequences AALN-pY-AQLMFP and AALN-pY-AQLWYA^56^ have similar dissociation constants for the N-SH2 domain (within a factor of 2), but the concentrations of these phosphopeptides needed for full activation of SHP2 differ by orders of magnitude. The interpretation of these studies is complicated by the fact that in principle these sequences could be dephosphorylated by SHP2, to different extents, during the activation experiments. Our data show that a concentration of the nonhydrolyzable phosphopeptide that almost completely saturates the N-SH2 domain causes only partial activation. While the inability of this specific sequence to favor activation cannot be ruled out, it is possible that partial activation is caused by inhibition of SHP2 activity due to association of the peptide with the PTP domain. In any case, even if activation of SHP2 without proper PPIs is inconsequential,^2,9,38^ it is important to note that the molecules developed here can inhibit the association of SHP2 with its partners, without causing complete activation, particularly for the wild-type protein.

Inhibition of PPIs, particularly using peptides, is currently a hot area of pharmaceutical research. For the RAS/MAPK pathway alone, at least 30 inhibitors of PPI have been developed, and several of them are undergoing clinical trials.^80^ However, no studies of this type have been reported in the case of SHP2, notwithstanding the central role of this phosphatase in the pathway. Peptides are particularly appealing for the inhibition of PPIs, where large interfaces are involved, which are difficultly targeted selectively by small molecules. An increasing number of drugs based on peptides or peptidomimetics is progressing in the drug development pipeline.^81^ Possible challenges in the therapeutic applications of peptide-based molecules are their rapid degradation and poor cell uptake, particularly for highly charged sequences.^81^ Here we successfully overcame the first hurdle, thanks to the introduction of non-natural amino acids. With regard to the second issue, several studies have demonstrated that efficient intracellular delivery of phosphopeptide mimics is possible, for instance, by conjugation to cell-penetrating sequences.^40,82−85^

Our *in vivo* findings on zebrafish embryos are very promising in light of potential applications, particularly considering that N-SH2 targeting peptides are more effective on activating mutants than on the wild-type protein, contrary to allosteric inhibitors such as SHP099.^23,25^ Indeed, in addition to their possible use as a research tool to study the role of PPIs in the function of SHP2, and regulation of the pathways controlled by this protein, including RAS/MAPK and PI3K/AKT signaling, the reported peptides constitute lead compounds for the development of new drugs against malignancies driven by *PTPN11* mutations, such as JMML, AMoL, and ALL, also considering that allosteric inhibitors have low activity against basally activated SHP2 variants.^23,25^ Finally, another possible field of therapeutic application is represented by rare diseases such as NS and NSML, which are caused by activating mutations of *PTPN11* (against which the available allosteric inhibitors are poorly active) and cause several severe postnatal, evolutive clinical manifestations, particularly hypertrophic cardiomyopathy.^15^

## Experimental Section

### Materials

Fmoc (9-fluorenylmethyloxycarbonyl)-amino acids were obtained from Novabiochem (Merck Biosciences, La Jolla, CA). Rink amide MBHA resin (0.65 mmol/g, 100–200 mesh) was purchased from Novabiochem. All other protected amino acids, reagents, and solvents for peptide synthesis were supplied by Sigma-Aldrich (St. Louis, MO). The LB medium components, all of the reagents used to prepare the buffers, and the Bradford reagent were purchased from Sigma-Aldrich. Tris(2-carboxyethyl)phosphine (TCEP) was obtained from Soltec Ventures (Beverly, MA). Spectroscopic grade organic solvents were purchased from Carlo Erba Reagenti (Milan, Italy). Cell culture medium growth factors and antibodies were purchased from VWR International PBI (Milan, Italy), EuroClone (Milan, Italy), Promega (Madison, WI), Invitrogen (Carlsbad, CA), Cell Signaling (Danvers, MA), Sigma-Aldrich, and Santa Cruz Biotechnology (Dallas, TX).

### Peptide Synthesis

The solid-phase peptide synthesis of the analogues described herein was performed on the Rink Amide MBHA resin, using standard Fmoc chemistry protocols. The phosphorylated amino acids Fmoc-Tyr(PO(OBzl)OH)-OH and Fmoc-Phe(CF_2_PO_3_)-OH are commercially available. The deprotection of the Fmoc group was performed with a 20% piperidine solution in *N*,*N*-dimethylformamide. The deprotection of the N^α^-function was carried out using a 20% piperidine solution in DMF, while for the activation of the carboxylic groups, a HBTU/HOBt mixture in a 2-fold molar excess was used in the presence of a 4-fold excess of DIPEA. On-resin N^α^-acetylation was achieved using an Ac_2_O/DIPEA mixture in DMF. For the fluorescent analogues, the introduction of the CF probe was carried out on resin and required the preactivation of 5(6)-carboxyfluorescein in the presence of HBTU, HOBT, and DIPEA, repeating the acylation step twice. The fluorescent analogues were obtained as a mixture of isomers. At the end of the synthesis, each peptide was cleaved from the resin using a mixture of TFA, TIS, and water in a 95:2.5:2.5 ratio. The filtrates were collected and concentrated under a flow of nitrogen, and the crude peptide was precipitated by addition of diethyl ether. The crude peptides were purified by flash chromatography on an Isolera Prime chromatographer (Biotage, Uppsala, Sweden) using a SNAP Cartridge KP-C18-HS 12g or preparative RP-HPLC on a Phenomenex C18 column (22.1 mm × 250 mm, 10 μm, 300 Å) using an Akta Pure GE Healthcare (Little Chalfont, U.K.) LC system equipped with an ultraviolet detector (flow rate of 15 mL/min) and a binary elution system: A, H_2_O; B, CH_3_CN/H_2_O [9:1 (v/v)]; gradient from 25% to 55% B in 30 min. The purified fractions were characterized by analytical HPLC-MS on a Phenomenex Kinetex XB-C18 column (4.6 mm × 100 mm, 3.5 μm, 100 Å) with an Agilent Technologies (Santa Clara, CA) 1260 Infinity II HPLC system and a 6130 quadrupole LC/MS instrument. High-resolution mass spectra were recorded using an ESI-Q-Tof *Micro* (Waters) spectrometer, in fast injection flow (50% ACN/water with 0.1% formic acid) and positive mode. As indicated in Table 1, all compounds were >95% pure, except for three compounds that had purities of 92–93% and were not used in biological testing.

Peptides were dissolved in DMSO to obtain 1–1.5 mM stock solutions. The exact concentration was obtained by ultraviolet (UV) measurements, exploiting the signal of CF for the labeled peptides and of pTyr, Tyr, and Trp for the unlabeled peptides. To this end, CF-labeled peptides were diluted from the stocks (1:100) in a buffer (pH 9), and their concentration was calculated from the CF signal at 490 nm using a molar extinction coefficient of 78000 M^–1^ cm^–1^.^86^ Unlabeled peptides were diluted 1:10 in a pH 7.4 buffer; molar extinction coefficients of Tyr, Phe, and Trp were taken from ref (87), while the molar extinction coefficient of pY was taken from ref (88).

### Protein Expression and Purification

The human esaHis-tagged *PTPN11* (residues 1–528) cDNA was cloned in a pET-26b vector (Novagen). Nucleotide substitutions associated with NS or leukemia were introduced by site-directed mutagenesis (QuikChange site-directed mutagenesis kit, Stratagene, San Diego, CA). A construct containing the cDNA encoding the isolated PTP domain preceded by the C-SH2 domain (residues 105–528) was generated by PCR amplification of the full-length wild-type cDNA and subcloned into the pET-26b vector (SHP2_Δ104_). A similar procedure was followed for the constructs of the N-SH2 (residues 2–111), C-SH2 (residues 109–217), and PTP (residues 212–528) domains and of the N-SH2/C-SH2 tandem (residues 2–217). Primer sequences are available upon request.

Recombinant proteins (wild type, mutants, and isolated domains) were expressed in *Escherichia coli* (DE3) Rosetta2 competent cells (Novagen), according to the following protocol described in ref (36). Briefly, following induction with isopropyl β-d-1-thiogalactopyranoside (Roche) (2 h at 30 °C or overnight at 18 °C), bacteria were centrifuged at 5000 rpm and 4 °C for 15 min. Bacterial pellets were resuspended in a lysozyme-containing lysis buffer [50 mM Tris-HCl (pH 8.5), 0.5 M NaCl, 20 mM imidazole, 1 mM tris(2-carboxyethyl)phosphine (TCEP), 100 mg/mL lysozyme, and one tablet of complete protease inhibitor cocktail] and sonicated in an ice bath, to avoid the heating of the sample. The lysate was centrifuged at 16000 rpm and 4 °C for 30 min. The supernatant was collected, and the protein of interest was purified by affinity chromatography on a Ni-NTA column (Qiagen, Hilden, Germany), using a buffer consisting of 50 mM Tris-HCl, 0.5 M NaCl, and 1 mM TCEP containing 20 mM imidazole for equilibration, 25 and 100 mM imidazole for washing, and 250 mM imidazole for elution. To remove imidazole, the samples were then dialyzed in a 20 mM Tris-HCl (pH 8.5) buffer containing 1 mM TCEP, 1 mM EDTA, and 50 mM NaCl (or 150 mM NaCl if no further purification steps followed). Full-length proteins and the SHP2_Δ104_ construct were then further purified by sequential chromatography, using an Äkta FPLC system (Äkta Purifier 900, Amersham Pharmacia Biotech, Little Chalfont, U.K.). The samples were first eluted within an anion exchange Hi-Trap QP 1 mL column (GE Helathcare, Pittsburgh, PA); the elution was carried out using 20 mM Tris-HCl (pH 8.5) in a NaCl gradient from 50 to 500 mM. The most concentrated fractions were then eluted in a gel filtration Superose column using 20 mM Tris-HCl buffer containing 150 mM NaCl as the mobile phase. Sample purity was checked by sodium dodecyl sulfate–polyacrylamide gel electrophoresis with Coomassie Blue staining and was always >90%. Proteins were quantitated by both the Bradford assay and the UV absorbance of aromatic residues, calculating extinction coefficients according to Pace.^87^ In general, the two methods were in agreement, but the values derived from UV absorbance were more precise and are reported in the figures and tables. The protein samples were used immediately after purification or stored at −20 °C and used within one week. In this case, after the samples had been thawed, 2.5 mM TCEP was added, the samples were centrifuged at 13000 rpm for 20 min, and the new concentration was re-evaluated. In the few cases in which the residual apparent absorbance due to light scattering was present in the UV spectra, it was subtracted according to Castanho.^89^

### Phosphatase Activity Assays

Catalytic activity was evaluated *in vitro* using 20 pmol of purified recombinant proteins in 200 μL of reaction buffer supplemented with 20 mM *p*-nitrophenyl phosphate (Sigma) as the substrate, either basally or following stimulation with the protein tyrosine phosphatase non-receptor-type substrate 1 (PTPNS1) bisphosphotyrosyl-containing motif (BTAM peptide) (GGGGDIT-pY-ADLNLPKGKKPAPQAAEPNNHTE-pY-ASIQTS) (Primm, Milan, Italy), as previously described.^22^ Proteins were incubated for 15 min (SHP2_Δ104_) or 30 min (SHP2) at 30 °C. Phosphate release was assessed by measuring the absorbance at 405 nm. Three replicates were performed for each measurement.

The ability of SHP2 to dephosphorylate the phosphopeptides was evaluated through a malachite green phosphatase assay (PTP assay kit 1, Millipore). The BTAM peptide and the following monophosphorylated peptides derived from known SHP2 substrates were used for comparison: DKQVE-pY-LDLDL (GAB1_Y657_), EEENI-pY-SVPHD (p190A/RhoGAP_Y1105_), and VDADE-pY-LIPQQ (EGFR_Y1016_) (Primm). SHP2_Δ104_ (2.4 pmol) was incubated with each phosphopeptide at 100 μM (total volume of 25 μL) for different times. The reaction was stopped by adding 100 μL of a malachite green solution. After 15 min, the absorbance was read at 655 nm, using a microplate reader, and compared with a phosphate standard curve to determine the release of phosphate. Data obtained in the linear region of the curve were normalized to the reaction time (1 min). Each experiment was performed in duplicate.

### Fluorescence Anisotropy Binding Assay

Anisotropy measurements were carried out using a Horiba Fluoromax 4 spectrofluorimeter. For the binding assays, the requested peptide amount (1 or 0.1 nM) was diluted in a buffer (10 mM HEPES, 150 mM NaCl, 1 mM EDTA, and 1 mM TCEP, fluorescence buffer henceforth) and its anisotropy signal was recorded. The peptide was then titrated with increasing protein amounts, until the anisotropy signal reached a plateau at its maximum value, or up to a protein concentration at which protein aggregation and consequent light scattering affected the anisotropy values (usually >1 μM). The measurements of CF-labeled peptides were carried out using an excitation wavelength of 470 nm and collecting the anisotropy values at an emission wavelength of 520 nm. A 495 nm emission filter was used. For the Cy3-labeled peptides, excitation and emission wavelengths of 520 and 560 nm were used. The lowest peptide concentration needed to have a sufficient fluorescent signal (0.1 nM) was used in the binding experiments. Higher concentrations (1 or 10 nM) were used for peptides with lower affinities and, therefore, higher *K*_d_ values. Between two and six replicates were performed (the specific numbers are indicated in the figures and in the table reporting the binding experiments).

The displacement assays were carried out with the same experimental settings. In this case, the labeled peptide–protein complex was titrated with increasing amounts of the unlabeled peptide, following the decrease in anisotropy. Measurements were carried out at the same CF-peptide concentration used for the corresponding binding experiments. With regard to the protein concentration, a compromise between two requirements is needed.^90^ On one hand, it is desirable to have a significant fraction of the CF-peptide bound to the protein, to maximize the dynamic range in the anisotropy signal, which decreases during the displacement experiment. On the other hand, the protein concentration should be comparable to or lower than the dissociation constant of the unlabeled peptide (*K*_d_^u^), to allow a quantitative and reliable determination of its binding affinity. Because several unlabeled peptides had a higher affinity than their fluorescent counterparts, in the displacement assays we used a protein concentration [P]_T_ that approximated *K*_d_, or in some cases even approximated *K*_d_/2 (where *K*_d_ is the dissociation constant for the labeled peptide). Between three and five replicates were performed (the specific numbers are indicated in the figures reporting the binding experiments). The equations used for data fitting are described in the Supporting Information.

### SH2 Domain Microarray

The microarray experiment was conducted by the Protein Array and Analysis Core at The University of Texas M. D. Anderson Cancer Center (Houston, TX), as previously described.^91^ Briefly, a library of SH2 domains^92^ was expressed as a GST fusion in *E. coli* and purified on glutathione-sepharose beads. The domains were spotted onto nitrocellulose-coated glass slides (Oncyte Avid slides, Grace Bio-Laboratories) using a pin arrayer. Each domain was spotted in duplicate. After incubation with a Cy3-P9W5 solution (0.5, 5.0, or 50 nM), fluorescence signals were detected using a GeneTACTM LSIV scanner (Genomic Solutions).

### *In Silico* Studies

#### System Preparation

The initial structure of the N-SH2 complexed with phosphopeptide P8 (Table 2) was obtained by amino acid substitutions (and deletions) in the crystallographic structure of the protein complexed with the GAB1 peptide (sequence of GDKQVE-pY-LDLDLD) (PDB entry 4QSY). The obtained complex was then used as the starting structure for subsequent amino acid substitutions in the bound peptide.

#### System Equilibration

MD simulations were performed using the GROMACS 2018.2 simulation package^93^ and a variant of AMBER99SB force field with parameters for phosphorylated residues.^94^ Water molecules were described by the TIP3P model. All of the simulated systems were inserted into a pre-equilibrated triclinic periodic box (15 nm × 7 nm × 7 nm), containing ∼24000 water molecules and counterions to neutralize the total charge of the system. They were relaxed first by doing a minimization with 5000 steepest descent cycles, by keeping protein positions fixed and allowing water and ions to adjust freely, followed by a heating protocol in which the temperature was progressively increased from 100 to 300 K. The system was then equilibrated for 100 ps in the *NVT* ensemble at 300 K, using velocity rescaling with a stochastic term (relaxation time of 1 ps)^95^ and then for 500 ps at a constant pressure (1 atm) using the Parrinello-Rhaman barostat (relaxation time of 5 ps). Long-range electrostatic interactions were calculated using the particle mesh Ewald method, and the cutoff distance for the nonbonded interaction was set to 12.0 Å. The LINCS constraint to all of the hydrogen atoms and a 2 fs time step were used.

#### Preparation of the Initial Configurations for Umbrella Sampling

For each system, a set of initial configurations was prepared by performing a center-of-mass (COM) pulling simulation. The distance between the peptide and N-SH2 domain COMs was constrained with a harmonic force (*K* = 1000 kJ mol^–1^ nm^–2^). Pulling was performed by gradually increasing the value of the equilibrium distance with a constant rate of 0.0025 nm/ps. The length of each simulation was ∼2.5 ns. During the whole simulation, a positional restraint (1000 kJ mol^–1^ nm^–2^) was applied to all heavy atoms in the N-SH2 domain except for atoms in loops around the binding region (residues 30–45, 52–75, and 80–100). For the choice of the optimal unbinding pathway, three different directions were tested, corresponding to (i) the vector from the phosphate to the α-carbon in pY, in the equilibrated complex; (ii) the vector defined by the initial positions of the two COMs; and (iii) the vector perpendicular to the surface of the cavity flanked by the EF and BG loops, passing through the N-SH2 domain center of mass. Among the three different pathways, the third direction encountered less steric occlusion by the EF and BG loops and was thus selected for further analyses.

#### Umbrella Sampling Simulations

A set of starting configurations was extracted from the pull-dynamics trajectory saving the peptide–protein center-of-mass distances every 2 Å in the range from 9 to ∼40 Å, thus affording ∼20 windows along the COM distance. The system in each window was preliminarily equilibrated for 1 ns with a strong positional restraint (1000 kJ mol^–1^ nm^–2^) to all α-carbon atoms except for those in loops flanking the binding region (as in the pull simulation), followed by a production run of 150 ns with the restraints. During this stage, a harmonic potential (*K* = 1000 kJ mol^–1^ nm^–2^) was applied on the distance between the two COMs. Additional sampling windows were added every ångström along the distance between the two COMs up to a distance of 15 Å. The resulting asymmetric distribution of sampling windows was used to calculate the PMF on the production run trajectories. The Weighted Histogram Analysis Method (WHAM) was used, with default settings (50 bins and tolerance of 10^–6^ kJ mol^–1^), using the gmx wham GROMACS tool. The analysis of the simulation was carried out on the 150 ns production dynamics, during which configurations were stored every 0.1 ns. The statistical uncertainty of the obtained PMF was estimated by bootstrapping analysis.^96^

### Peptide Stability in Serum and in DMEM

The peptides were dissolved in DMSO (5 mg/mL). In Eppendorf tubes, 1 mL of HEPES buffer (25 mM, pH 7.6) was temperature equilibrated at 37 °C before the addition of 250 μL of human serum and 20 μL of a peptide solution; the reaction was followed for 24 h. At fixed intervals, 100 μL of the solution was withdrawn and added to 200 μL of absolute ethanol. These samples were kept on ice for 15 min and then centrifuged at 13000 rpm for 5 min; the supernatant solutions were analyzed by HPLC and HPLC-MS with a 20% to 60% B gradient in 20 min to follow the reaction. In parallel, samples containing peptide, buffer, and ethanol only were analyzed. A degradation resistance test was also conducted in DMEM (Dulbecco’s modified Eagle’s medium). The experimental conditions are similar to those described above; the reaction was followed for 72 h. The enzymatic degradation resistance tests were followed by HPLC using a 5% to 50% B gradient over 20 min.

### *In Vivo* Zebrafish Rescue Experiments

One-cell stage zebrafish embryos were injected with a mixture of 120 ng/μL mRNA encoding either GFP-2A-Shp2-D61G or GFP-2A-Shp2-wild-type (as a control), with or without OP, at final concentrations of 0.3, 3, and 5 μM (obtained by injecting ∼1 nL of a concentrated solution and assuming a volume of ∼500 nL for the embryo).^97^ Embryos were selected on the basis of proper GFP expression and imaged at 11 hpf in their lateral position using the Leica M165 FC stereomicroscope. Images were analyzed using ImageJ,^98^ by measuring the ratio of the major and minor axis from a minimum of 31 embryos (the number of embryos used for each specific experiment is reported in the figure reporting these data). Statistical analysis was performed in GraphPad Prism, using the analysis of variance (ANOVA) complemented by Tukey’s honest significant difference test (Tukey’s HSD). To measure the survival of injected embryos, a minimum of 48 embryos per group were grown up to 4 days postfertilization and counted on days 1 and 4. Survival curves were plotted using GraphPad Prism, and the differences between samples were determined using the log rank (Mantel-Cox) test. The number of embryos used for each specific experiment is indicated in the figure reporting these data.

## Supporting Information Available

Methods (with the derivation of the equation used in the analysis of the binding curves), binding selectivity of CF-P9W5 for the two SH2 domains of SHP2 (Figure S1), binding of peptide Cy3-P9W5 to the N-SH2 domain (Figure S2), binding of the nondephosphorylatable peptides CF-OP and OP to the N-SH2 domain (Figure S3), resistance of OP and P8W5 to proteolytic degradation in human serum (Figure S4), dephosphorylation of P8W5 and other phosphopeptides by SHP2_Δ104_ (Figure S5), association of CF-OP with the PTP domain (Figure S6), representative images of zebrafish embryos 11 hpf (Figure S7), HPLC profile for the OP peptide (Figure S8), and literature values of the IRS-1 pY1172/N-SH2 domain dissociation constant (Table S1) (PDF)
Molecular formula strings (CSV)

## Abbreviations Used

ALL: acute lymphoblastic leukemia
AMoL: acute monocytic leukemia
CagA: cytotoxicity-associated immunodominant antigen
CF: carboxyfluorescein
Cy3: cyanine 3 carboxylic acid
F_2_Pmp: phosphonodifluoromethyl phenylalanine
ST: glutathione S-transferase
hpf: hours post fertilization
JMML: juvenile myelomonocytic leukemia
NS: Noonan syndrome
NSML: Noonan syndrome with multiple lentigines
PDB: Protein Data Bank
PPI: protein–protein interaction
PTK: protein tyrosine kinase
PTP: protein tyrosine phosphatase
pY: phosphotyrosine
RTK: receptor tyrosine kinase
SH2: Src homology 2
SHP2: SH2 domain-containing phosphatase 2
shRNA: short hairpin ribonucleic acid.
