# Supplementary material for: Targeting Oncogenic Src Homology 2 Domain-Containing Phosphatase 2 (SHP2) by Inhibiting Its Protein–Protein Interactions
Source: J Med Chem. 2021 Oct 29;64(21):15973–90. doi: 10.1021/acs.jmedchem.1c01371 (PMC8591604; doi:10.1021/acs.jmedchem.1c01371)
Supplement: Supplementary file 1 — jm1c01371_si_001.pdf [file jm1c01371_si_001.pdf]

Supporting information

Targeting Oncogenic Src Homology 2 Domain-Containing Phosphatase 2 (SHP2) by Inhibiting its Protein-Protein Interactions

Sara Bobone,[1] Luca Pannone,[2,3] Barbara Biondi,[4] Maja Solman,[5] Elisabetta Flex,[3] Viviana Claudia Canale,[1] Paolo Calligari,[1] Chiara De Faveri,[6] Tommaso Gandini,[6] Andrea Quercioli,[1] Giuseppe Torini,[1] Martina Venditti,[2] Antonella Lauri,[2] Giulia Fasano,[2] Jelmer Hoeksma,[5] Valerio Santucci,[1] Giada Cattani,[1] Alessio Bocedi,[1] Giovanna Carpentieri,[2,3] Valentina Tirelli,[7] Massimo Sanchez,[7] Cristina Peggion,[6] Fernando Formaggio,[4,6] Jeroen den Hertog,[4,8] Simone Martinelli,[3,†] Gianfranco Bocchinfuso,[1,†] Marco Tartaglia,[2,†] Lorenzo Stella[1,*]

1. Department of Chemical Science and Technologies, University of Rome Tor Vergata, Rome, 00133, Italy.

2. Genetics and Rare Diseases Research Division, Ospedale Pediatrico Bambino Gesù, IRCCS, Rome, 00146, Italy.

3. Dipartimento di Oncologia e Medicina Molecolare, Istituto Superiore di Sanità, Rome, 00161, Italy.

4. Institute of Biomolecular Chemistry, Padova Unit, CNR, Padova, 35131, Italy.

5. Hubrecht institute - KNAW and University Medical Center Utrecht, Utrecht, 3584 CT, The Netherlands.

6. Department of Chemical Sciences, University of Padova, Padova, 35131, Italy.

7. Centre of Core Facilities, Istituto Superiore di Sanità, Rome, 00161, Italy.

8. Institute of Biology Leiden, Leiden University, Leiden, 2333 BE, the Netherlands.

* To whom correspondence should be addressed:

E-mail: stella@stc.uniroma2.it

Contents

Supplementary Materials and Methods, with the derivation of the equations used in the analysis of the binding curves;

Figures S1: binding selectivity of CF-P9W5 for the two SH2 domains of SHP2;

Figure S2: binding of the peptide Cy3-P9W5 to the N-SH2 domain;

Figure S3: binding of the non-dephosphorylatable peptides CF-OP and OP to the N-SH2 domain;

Figure S4: resistance of OP and P8W5 to proteolytic degradation in human serum;

Figure S5: dephosphorylation of P8W5 and other phosphopeptides by $SHP2_{\Delta104}$;

Figure S6: CF-OP association to the PTP domain;

Figure S7: representative images of zebrafish embryos at 11 hours postfertilization;

Figure S8: HPLC profile for the OP peptide;

Table S1: Literature values for IRS-1 pY1172/N-SH2 domain dissociation constant.

Molecular Formula Strings

Supplementary materials and methods

Analysis of binding curves

Fluorescence anisotropy is an additive quantity and the free and bound form of a labeled peptide contribute to the measured anisotropy proportionally to the fraction of the total fluorescence they emit. Since, in our case, the fluorescence intensity does not change appreciably upon binding, these fractions correspond to the population of the two states.

$$r = x_B r_{max} + (1 - x_B) r_0 \qquad (1)$$

Here, r, r_0 and r_{max} are the anisotropy values at a given protein concentration, in the absence of protein and when the peptide is completely bound, respectively, and x_B is the molar fraction of bound peptide. This last quantity, therefore, can be derived from the anisotropy values as follows:

$$x_B = \frac{r - r_0}{r_{max} - r_0} \qquad (2)$$

It is worth mentioning that the fluorescence polarization parameter (often used in place of fluorescence anisotropy) is not additive and therefore in that case the equation for the determination of the bound fraction is more complex.[1]

When saturation conditions could not be attained, due to relatively high values of the dissociation constant (in particular for the CF-P9Y0/N-SH2 binding curve reported in Figure 2), r_{max} was derived by a binding curve corresponding to a higher affinity interaction (the CF-P9/N-SH2 data), since this parameter is determined essentially by the fluorophore's properties and by the protein/domain hydrodynamic volume, which are the same in both cases.

K_d values were obtained fitting the data with the following equation,[2] which avoids the need for the commonly used (but often unjustified) approximation of the concentration of unbound protein with the total concentration:

$$\frac{r - r_0}{r_{max} - r_0} = \frac{[P]_T + [L]_T + K_d - \sqrt{([P]_T + [L]_T + K_d)^2 - 4[P]_T[L]_T}}{2[L]_T} \qquad (3)$$

Here, $[P]_T$ and $[L]_T$ are the total protein and ligand concentrations. When allowed by the experimental conditions (i.e. when $[L]_T \ll K_d$), this equation was simplified by assuming $[P]_T \cong [P]$ and obtaining:

$$\frac{r - r_0}{r_{max} - r_0} = \frac{{[P]_t}/{K_d}}{1 + {[P]_t}/{K_d}} \qquad (4)$$

The affinity of unlabeled peptides was determined by competition experiments, in which a sample with fixed total protein and fluorescently labeled peptide concentrations ($[P]_T$ and $[L]_T$) was titrated with the unlabeled peptide, causing displacement of the fluorescent peptide and a decrease in anisotropy. From these data, the EC_{50} (*i.e.* the total concentration of unlabeled peptide that displaces half of the bound fluorescent analog) was determined, interpolating the displacement curve using a phenomenological Hill equation:[3]

$$\frac{r - r_0}{r_{fin} - r_0} = \frac{\left\{{[I]_T}/{EC_{50}}\right\}^n}{1 + \left\{{[I]_T}/{EC_{50}}\right\}^n} \qquad (5)$$

where $[I]_T$ is the total concentration of the peptide causing the displacement, and r_{fin} is the anisotropy corresponding to total displacement, while in this case r_0 is the starting anisotropy, in the absence of displacing peptide.

Successively, the dissociation constant of the unlabeled peptide (K_d^u) was calculated from the know values of EC_{50}, K_d, $[P]_T$ and $[L]_T$, as described here below. Our treatment follows that of Nikolovska-Coleska,[4] through a slightly simplified route, and correcting some inaccuracies present in the equations of that article.

In the system where protein (P), ligand (L) and a competitive inhibitor (I) are present, both L and I can form complexes with P (PL and PI, respectively). The following dissociation constants can be defined for the two binding equilibria:

$$K_d = \frac{[P][L]}{[PL]};\ \ K_d^u = \frac{[P][I]}{[PI]} \qquad (6)$$

and the following mass conservation laws apply:

$$[P]_T = [P] + [PI] + [PL];\ [L]_T = [L] + [PL];\ [I]_T = [I] + [PI] \qquad (7)$$

Let’s define $[PL]_0$ as the complex concentration in the absence of inhibitor. Then, by definition, at the EC_{50}

$$[PL]_{50} = \frac{[PL]_0}{2} \qquad (8)$$

At the EC_{50},

$$\frac{[PL]_{50}}{[P]_T} = \frac{[PL]_{50}}{[P]_{50} + [PL]_{50} + [PI]_{50}} = \frac{1}{1 + \frac{[P]_{50}}{[PL]_{50}} + \frac{[PI]_{50}}{[PL]_{50}}} = \frac{1}{1 + \frac{[P]_{50}}{[PL]_{50}}\left(1 + \frac{[PI]_{50}}{[P]_{50}}\right)} \qquad (9)$$

and therefore

$$[PL]_{50} = \frac{[PL]_0}{2} = \frac{[P]_T}{1 + \frac{K_d}{[L]_{50}}\left(1 + \frac{[I]_{50}}{K_d^u}\right)} \qquad (10)$$

This equation can be inverted to calculate K_d^u

$$K_d^u = \frac{[I]_{50}}{\left(\frac{2[P]_T}{[PL]_0} - 1\right)\frac{[L]_{50}}{K_d} - 1} \qquad (11)$$

For $[L]_{50}$ we can write:

$$[L]_{50} = [L]_T - [PL]_{50} = [L]_T - \frac{[PL]_0}{2} \quad (12)$$

Finally, for $[I]_{50}$ we can write:

$$[I]_{50} = EC_{50} - [PI]_{50} = EC_{50} - ([P]_T - [P]_{50} - [PL]_{50}) =$$

$$= EC_{50} - [P]_T + K_d\frac{[PL]_{50}}{[L]_{50}} + [PL]_{50} = EC_{50} - [P]_T + [PL]_{50}\left(1 + \frac{K_d}{[L]_{50}}\right) =$$

$$= EC_{50} - [P]_T + \frac{[PL]_0}{2}\left(1 + \frac{K_d}{[L]_T - \frac{[PL]_0}{2}}\right) \qquad (13)$$

Substituting the above equations in the expression for K_d^u, we get:

$$K_d^u = \frac{EC_{50} - [P]_T + \frac{[PL]_0}{2}\left(1 + \frac{K_d}{[L]_T - \frac{[PL]_0}{2}}\right)}{\left(\frac{2[P]_T}{[PL]_0} - 1\right)\frac{[L]_T - \frac{[PL]_0}{2}}{K_d} - 1} \quad (14)$$

Finally, $[PL]_0$ can be substituted with the following expression, analogous to Eq. (3):

$$[PL]_0 = \frac{[P]_T + [L]_T + K_d - \sqrt{([P]_T + [L]_T + K_d)^2 - 4[P]_T[L]_T}}{2} \quad (15)$$

In this way, K_d^u is expressed as a function of the known quantities EC_{50}, K_d, $[P]_T$ and $[L]_T$, without any approximation.

Supplementary figures

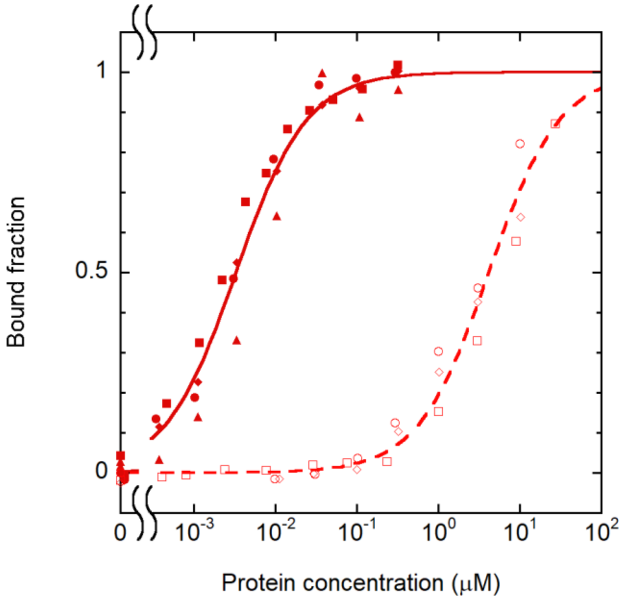

Figure S1: binding selectivity of CF-P9W5 for the two SH2 domains of SHP2

Comparison of the association curves of CF-P9W5 to the N-SH2 and C-SH2 domains of SHP2. Experimental conditions for the N-SH2 binding experiments: see Fig. 4; for the C-SH2 binding experiments: [CF-P9W5] = 1.0 nM. Independent, replicate experiments (n=4 for N-SH2, n=3 for C-SH2) are reported with different symbols and were fit collectively.

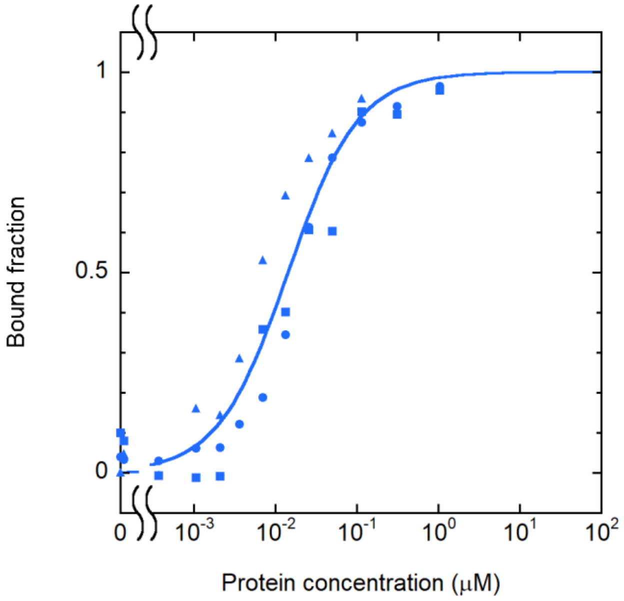

Figure S2: binding of the peptide Cy3-P9W5 to the N-SH2 domain.

[Cy3-P9W5]=1.0 nM. Independent, replicate experiments (n=3) are reported with different symbols and were fit collectively.

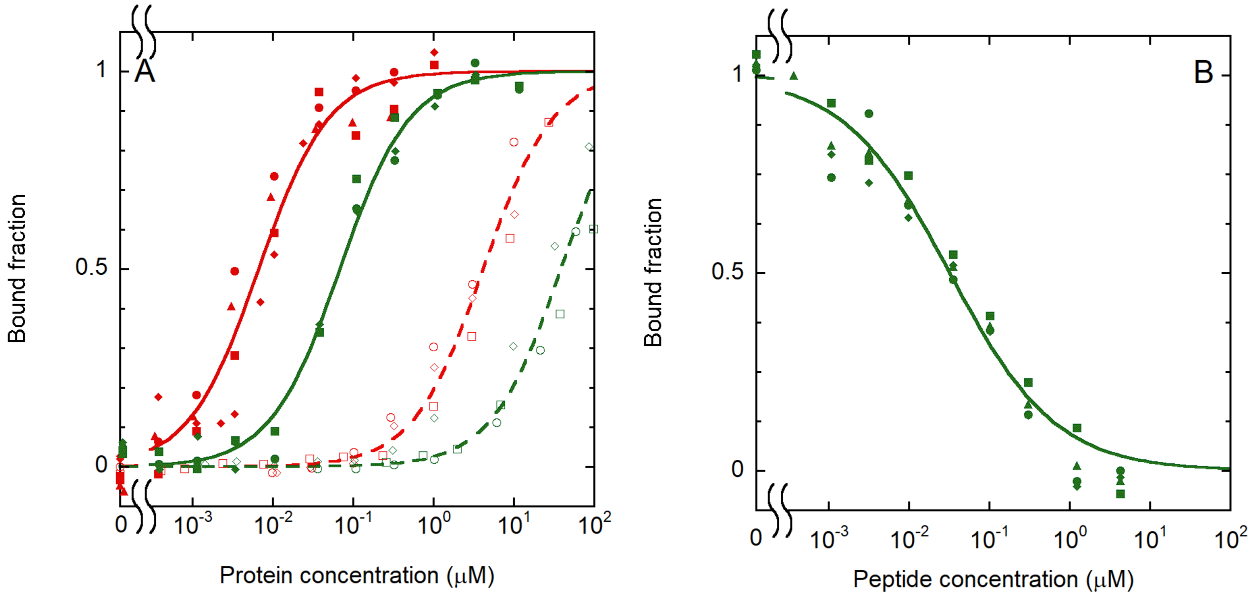

Figure S3: binding of the non-dephosphorylatable peptides (CF-OP and OP) to the N-SH2 and C-SH2 domains.

A) binding curves for CF-OP (green). For comparison, the curves for CF-P9W5 are also shown (red). Full symbols, solid lines: N-SH2 domain; empty symbols, dashed lines: C-SH2 domain. [CF-OP]=1.0 nM, for binding to the N-SH2 domain, 10 nM for binding to the C-SH2 domain. For the experimental conditions used in the CF-P9W5 experiments, see Figure S1.

B) displacement curve for the unlabeled analog OP and the N-SH2 domain. The bound fraction of labeled peptide is reported as a function of the concentration of competing, unlabeled peptide. [CF-P9W5] = 0.5nM, [N-SH2] = 3.4 nM.

Independent, replicate experiments (n=3) are reported with different symbols and were fit collectively.

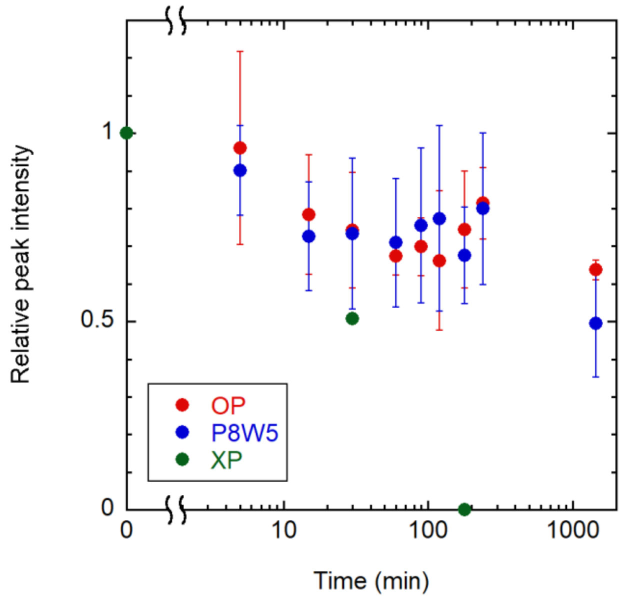

Figure S4. resistance of OP and P8W5 to proteolytic degradation in human serum

After incubation with human serum for different times, the peptides were analyzed by HPLC. The integral of the elution peak is reported as a function of time. The experiment was performed in triplicate. Error bars represent standard deviations. By comparison, degradation of the peptide XP (WFKYYGKAIY, with free termini) [Reference 60 in the main text], used as a positive control, is reported (n=1).

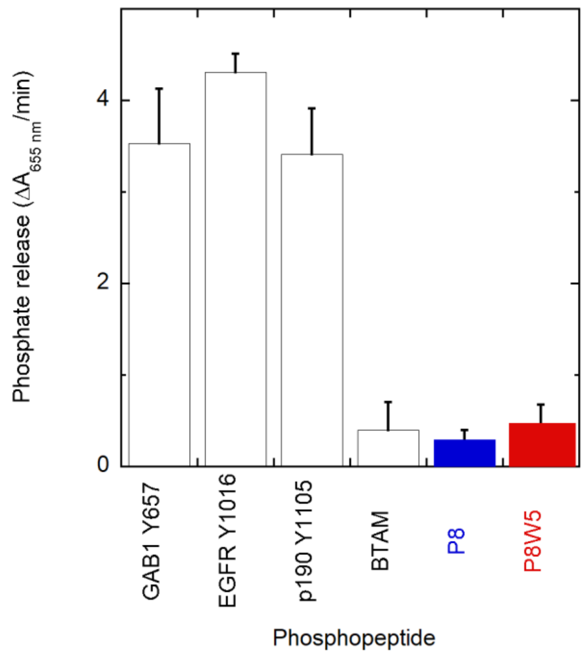

Figure S5: dephosphorylation of P8W5 and other phosphopeptides by SHP2$_{\Delta 104}$.

Dephosphorylation of various phosphopeptides by a constitutively active SHP2 construct, lacking the N-SH2 domain (i.e. the first 104 residues, SHP2$_{\Delta 104}$). In addition to P8 and P8W5, the following phosphopeptides were used for comparison:

GAB1 Y657 (DKQVE-pY-LDLDL)

p190A/RhoGAP Y1105 (EEENI-pY-SVPHD)

EGFR Y1016 (VDADE-pY-LIPQQ)

BTAM, or bisphosphorylated SHSP-1 TAM1 (GGGGDIT-pY-ADLNLPKGKKPAPQAAEPNNHTE-pY-ASIQTS, with 4 N-terminal G residues)

SHP2$_{\Delta 104}$ was used at a 95 nM concentration. Phosphopeptides were added at a 100 μM concentration and the phosphate released was measured at different times. From the linear region of the phosphate versus time curve, the variation in absorbance at 655 nm in 1 min, due to phosphate release, was calculated and plotted. Each experiment was performed in duplicate.

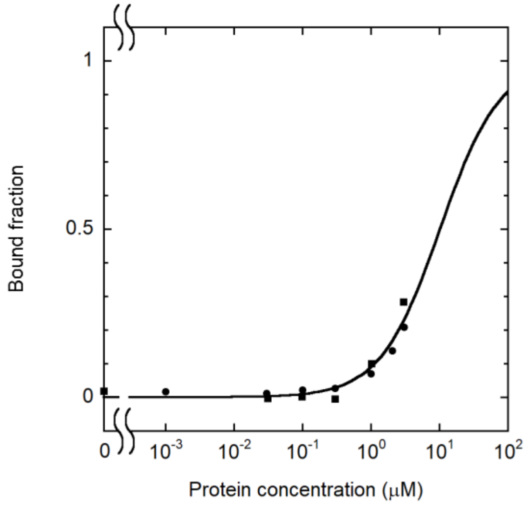

Figure S6. CF-OP association to the PTP domain.

[CF-P9ND0W5]=1.0 nM. Independent, replicate experiments (n=2) are reported with different symbols and were fit collectively.

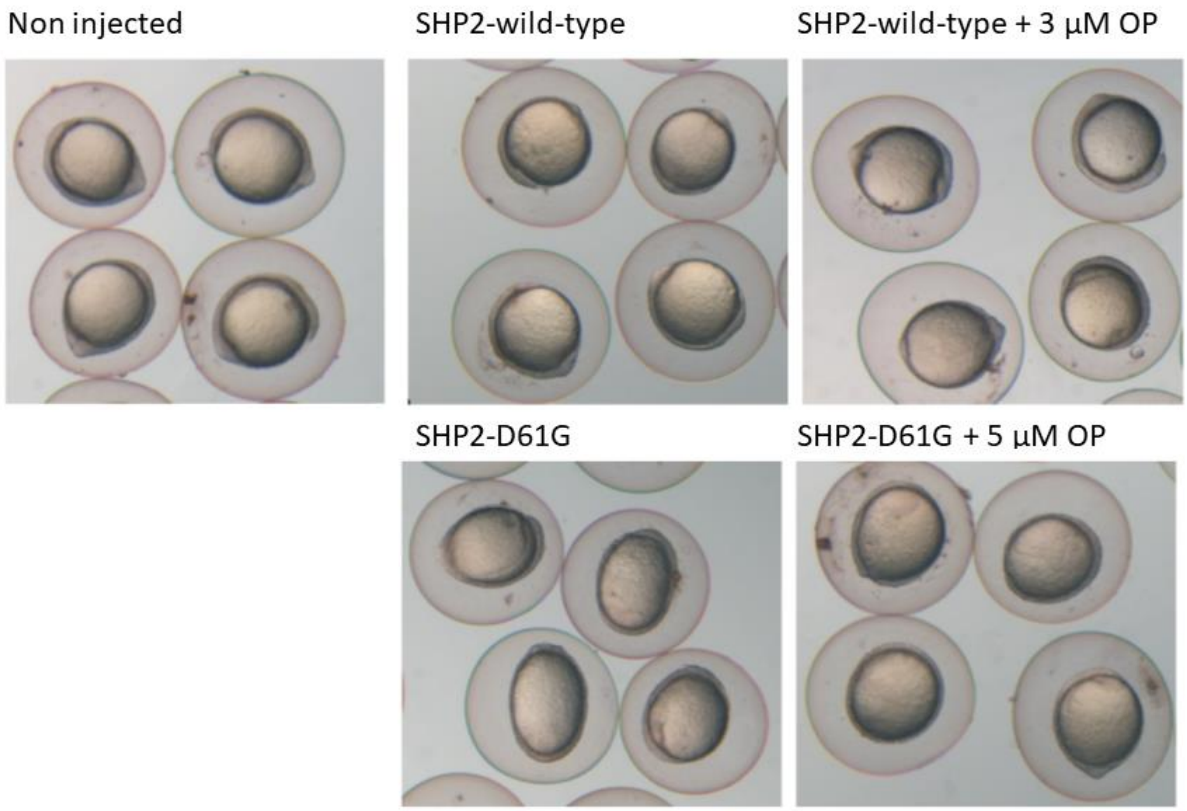

Figure S7: representative images of zebrafish embryos at 11 hours postfertilization.

The ovality in D61G embryos is rescued by the OP peptide, in a dose-dependent manner. Embryos were injected at the one-cell stage with mRNA encoding GFP-2A-Shp2-D61G or GFP-Shp2-wild-type with or without the OP peptide at 0.3 µM, 3 µM and 5 µM concentration. Non-injected embryos (ni) were evaluated as a control.

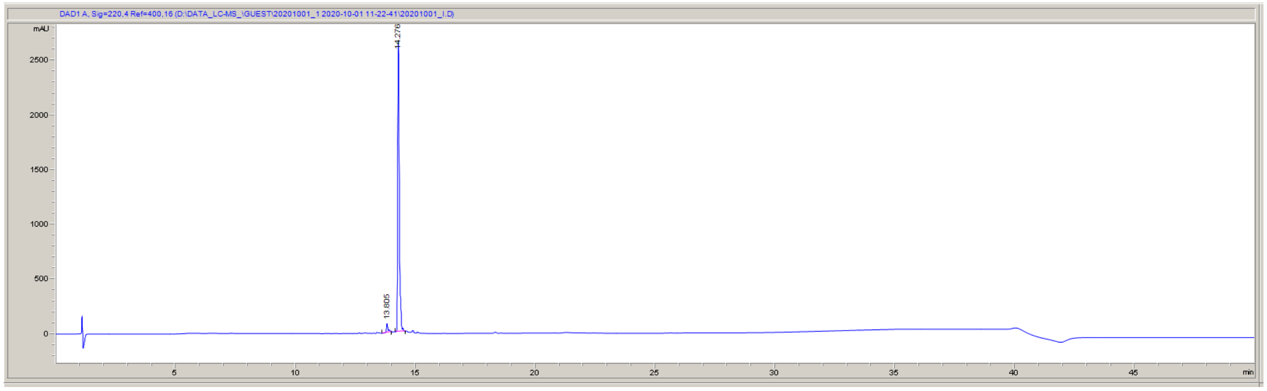

Figure S8: HPLC profile for the OP peptide.

Supplementary tables

Table S1. Literature values for IRS-1 pY1172/N-SH2 domain dissociation constant.

Reference<br><br>(numbers refer to the main text)	Method	K_d
48	Radioactively labeled peptide	~1-10 μM
49	Surface plasmon resonance	14±8 nM
19	Isothermal titration calorimetry	51 nM

Supplementary references

1) Jameson, D. M. Introduction to fluorescence. Taylor & Francis Eds, Boca Raton, **2014**.

2) Van de Weert, M.; Stella, L. Fluorescence quenching and ligand binding: a critical discussion of a popular methodology. *J. Mol. Struct.*, **2011**, *998*, 144-150.

3) Barlow, R.; Blake, J. F. Hill coefficients and the logistic equation. *Trends Pharmacol. Sci.*, **1989**, *10*, 440-441.

4) Nikolovska-Coleska, Z.; Wang, R.; Fang, X.; Pan, H.; Tomita, Y.; Li, P.; Roller, P.P.; Krajewski, K.; Saito, N.G.; Stuckey, J.A. Wang, S. Development and optimization of a binding assay for the XIAP BIR3 domain using fluorescence polarization. *Anal. Biochem*. **2004**, *332*, 261-273.
